# Superconductivity Above 100 K Predicted in Carbon‐Cage Network

**DOI:** 10.1002/advs.202303639

**Published:** 2023-10-09

**Authors:** Yu‐Long Hai, Meng‐Jing Jiang, Hui‐Li Tian, Guo‐Hua Zhong, Wen‐Jie Li, Chun‐Lei Yang, Xiao‐Jia Chen, Hai‐Qing Lin

**Affiliations:** ^1^ Shenzhen Institute of Advanced Technology Chinese Academy of Sciences Shenzhen 518055 China; ^2^ Nano Science and Technology Institute University of Science and Technology of China Suzhou 215123 China; ^3^ University of Chinese Academy of Sciences Beijing 100049 China; ^4^ School of Science Harbin Institute of Technology Shenzhen 518055 China; ^5^ Center for High Pressure Science and Technology Advanced Research Shanghai 201203 China; ^6^ School of Physics Zhejiang University Hangzhou 310058 China

**Keywords:** cage‐network, clathrate carbide, electron–phonon coupling, electronic structures, first‐principles, superconductivity

## Abstract

To explore carbide superconductors with higher transition temperature, two novel carbon structures of cage‐network are designed and their superconductivity is studied by doping metals. *M*C_6_ and *M*C_10_ are respectively identified as C_24_ and C_32_ cage‐network structures. This study finds that both carbon structures drive strong electron–phonon interaction and can exhibit superconductivity above liquid nitrogen temperature. Importantly, the superconducting transition temperatures above 100 K are predicted to be achieved in C_24_‐cage‐network systems doped by Na, Mg, Al, In, and Tl at ambient pressure, which is far higher than those in graphite, fullerene, and other carbides. Meanwhile, the superconductivity of cage‐network carbides is also found to be sensitive to the electronegativity and concentration of dopant *M*. The result indicates that the higher transition temperatures can be obtained by optimizing the carbon‐cage‐network structures and the doping conditions. The study suggests that the carbon‐cage‐network structure is a direction to explore high‐temperature superconducting carbides.

## Graphical abstract

The novel carbon structures of cage‐network is designed. The superconducting transition temperature above 100 K are predicted to be achieved in C_24_‐cage‐network systems doped by metals at ambient pressure, which is far higher than those in graphite, fullerene, and other carbides. The study suggests that the carbon‐cage‐network structure is a direction to explore high‐temperature superconducting carbides.

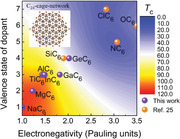

## 1 Introduction

Superconductivity has always been one of the most fascinating research topics in condensed matter physics. Recently, near room‐temperature superconductivity has been observed in metal hydride at high pressure above 100 GPa,^[^ ^1^, ^2^ ^]^ which attracts much attention to the research field of light‐weight element high‐temperature superconductors. Compared with hydrides, carbides are another widely concerned materials. As we all know, carbon is a magical element and widely exists in our life. With the different hybridization forms of *sp* ^3^, *sp* ^2^, and *sp* between carbon atoms, different structures and morphologies can be formed such as diamond, graphite, nanotube, and fullerene, *etc*. Significantly, carbon‐based materials were also suggested to be one of the most promising high‐temperature or room‐temperature superconductors,^[^ ^3^ ^]^ which can play important roles in energy, information, electronics, medical, and other fields. Superconductivities of different carbon structures have been explored experimentally and theoretically. However, the superconducting transition temperature (*T*_*c*_) of carbide is far from the expected value. For example, the *T*_*c*_ in boron‐doped diamond is only about 4 K,^[^ ^4^ ^]^ although the boron content increases, the *T*_*c*_ can reach about 55 K.^[^ ^5^ ^]^ For carbon structure with layered characteristics, the highest *T*_*c*_ of metal inserted graphite is 11.5 K (CaC_6_).^[^ ^6^ ^]^ Recently, the unconventional superconductivity in magic‐angle graphene superlattices has rekindled interest of carbon‐based materials,^[^ ^7^, ^8^ ^]^ but the *T*_*c*_ from the resistance measurement is still below a few Kelvins. In Li‐decorated monolayer graphene, the *T*_*c*_ is only characterized as 5.9 K.^[^ ^9^ ^]^ For 1D system, single‐walled carbon nanotube (SWCNT) with a length of 4 Å exhibits the superconductivity of 15 K.^[^ ^10^ ^]^ And the *T*_*c*_ in boron‐doped SWCNT is only 12 K.^[^ ^11^ ^]^ For fullerene (C_60_) crystal, the superconductivity was discovered and the *T*_*c*_ can be tuned in the range of 18 − 40 K in alkali metal‐doped C_60_.^[^ ^12^, ^13^ ^]^ Some clathrate carbon structures similar to C_60_ were also investigated. For instance, the *T*_*c*_ can be reached 55 K in Na‐doped C_20_.^[^ ^14^ ^]^ And it was predicted that the superconductivity is decreased with the increase of the size of carbon cage.^[^ ^15^ ^]^ In a carbide CrC with the rocksalt structure, the superconductivity of 35 K was predicted.^[^ ^16^ ^]^ Compared with the highly concerned hydride superconductors nowadays, the *T*_*c*_ of the aforementioned carbides is generally relatively low. However, the superconductivity of these carbides is almost achieved at ambient pressure, unlike hydrides, which often require a pressure of over 100 GPa. Therefore, in exploring the high‐temperature superconductivity at ambient pressure, carbides have more advantages than hydrides. At present, the main reason for the low *T*_*c*_ in carbides is that the optimal crystal structure formed by carbon atoms has not been found. For example, the near room‐temperature superconductivity of hydrides is almost only present in some special structures, such as cage configuration.^[^ ^17^, ^18^ ^]^ Hence, it is very important to explore novel structures for the high‐temperature superconductivity of carbides.

From previous studies, the *T*_*c*_ of carbide is usually <50 K. By investigating the structural characteristics, we can see that C_60_ molecules are isolated in the fullerene crystal, carbon nanotubes are isolated from each other in the plane direction, and the coupling of graphene in the interlayer direction is prohibited. So we speculate that one of possible reasons of the low *T*_*c*_ is the lack of strong structural coupling between carbon structural units, which leads to relatively low superfluid density. Namely, the strong structural coupling may drive the superconductivity with higher *T*_*c*_. In a Q‐carbon structure, the superconductivity of *T*_*c*_ = 55 K was observed.^[^ ^19^, ^20^ ^]^ In the clathrate carbon structure of FC_34_ with the coalescence feature of big and small cages, the *T*_*c*_ was theoretically predicted as 77 K.^[^ ^21^ ^]^ The predicted *T*_*c*_ can be close to 80 K in metal‐doped (CB)_3_ clathrates.^[^ ^22^, ^23^, ^24^ ^]^ Additionally, in clathrate carbides with sodalite structure, the *T*_*c*_ was predicted to exceed 100 K.^[^ ^25^, ^26^, ^27^ ^]^ These reported results imply the importance of structural bond coupling among carbon cluster units. Furthermore, it was found that the clathrate structure is a good material gene and more likely to produce the high superconducting transition temperature in carbides. Of course, it is better to have strong coupling between cages rather than isolated like C_60_. Here, we design two carbon cages of C_24_ and C_32_ by referring to the clathrate structures formed by H atoms in CaH_6_^[^ ^28^ ^]^ and LaH_10_.^[^ ^17^ ^]^ The basic principle of structural design is that carbon atoms form obvious cage‐like features, and there are obvious coupling characteristics between the cages, such as through C‐C bonds or shared atomic surfaces. Under the symmetry of the space group, the carbon‐cage‐network structure‐based on C_24_ or C_32_ cage is formed, respectively. The superconductivity above 100 K is predicted in the carbon‐cage‐network structures.

## 2 Results and Discussion

**Figure 1:**
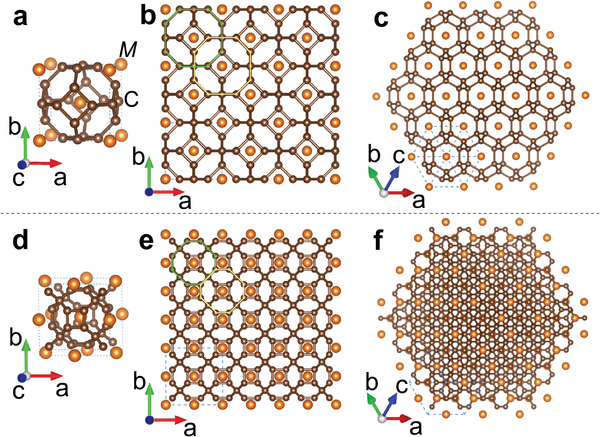
The sketch of carbon‐cage‐network doped by metal. a–c) are corresponding to *M*C_6_ with $Im\bar{3}m$ space‐group, while (d–f) are corresponding to *M*C_10_ with $Fm\bar{3}m$ space‐group. (a) and (d) is the unit cell of *M*C_6_ and *M*C_10_, respectively. Others are supercell configurations.

**Figure** **1** shows the geometric characteristics of two carbon structures‐doped by metal. Their chemical formulas can be expressed as *M*C_6_ and *M*C_10_ respectively, where *M* represents the doped metal. As shown in Figure 1a, *M*C_6_ possesses the sodalite‐structure with $Im\bar{3}m$ space‐group. The unit cell of *M*C_6_ contains two metal atoms and twelve carbon atoms. A cage made of 24 carbon atoms is formed in *M*C_6_, calling as C_24_ cage, and it is composed of six quadrilaterals and eight hexagons. Remarkably, seen from the aspect of supercell, these C_24_ cages are connected by sharing quadrilateral rings in each axial direction, while these cages are overlapping along the diagonal direction of the plane, such as those marked in green and yellow lines in Figure 1b. As a result, these carbon cages forms a 3D network, with doping metal in the center of each cage. This kind of carbon‐cage network in the whole crystal is not only different from the isolated feature of cage in fullerenes^[^ ^12^, ^13^ ^]^ and some so‐called smaller fullerenes such as C_20_,^[^ ^14^ ^]^ C_28_,^[^ ^29^ ^]^ and C_36_,^[^ ^30^ ^]^ but also different from the coalescence of big and small cages in FC_34_,^[^ ^21^ ^]^ LiC_40_,^[^ ^31^ ^]^ and Li_8_C_46_.^[^ ^32^ ^]^ For *M*C_10_ as shown in Figure 1d, it is another kind of sodalite‐like structure with $Fm\bar{3}m$ space‐group. The unit cell of *M*C_10_ consists of four metal atoms and forty carbon atoms, in which a cage made of 32 carbon atoms is formed, calling as C_32_ cage. Each C_32_ cage is composed of six quadrilaterals and twelve hexagons. Similar to *M*C_6_, *M*C_10_ also exhibits the feature of carbon‐cage network as shown in Figure 1e. But C_32_ cages are connected by sharing hexagon rings along the diagonal direction of the plane, such as those marked in green and yellow lines in Figure 1e, and these C_32_ cages are overlapping in each axial direction. Additionally, the viewing from the aspect shown in Figure 1c,f displays the difference between *M*C_6_ and *M*C_10_. Comparing C_24_ with C_32_ cage, we find that the diameter of the former is smaller than that of the latter. In C_24_ cage, all the lengths of the nearest neighbor C‐C bonds are uniform, while in C_32_ cage, there are two kinds of the lengths of the nearest neighbor C‐C bonds. The C‐C bonding length forming hexagonal ring is less than that forming quadrilateral ring in C_32_ cage.

Based on C_24_ and C_32_ cage‐network structures, we have considered the situation that most of the metals in the periodic table are doped into these two carbon structures. The structural optimization, electronic structures, and phonon spectra were calculated based on the first‐principles method. When the doping of a metal satisfies both metallization and dynamical stability, we consider it a candidate system for investigating superconductivity. Table S1 (Supporting Information) presents the evaluation of metallization and dynamical stability for metal‐doped two carbides with cage‐network configuration. To compare with previous predictions,^[^ ^25^, ^26^, ^27^ ^]^ the pressure factor was also considered in our calculations. It is found that two cage‐network structures doped by metals are good candidates. The density of states (DOS) shown in Figures S1– S29 (Supporting Information) and phonon spectra without imaginary frequency in Figures S30– S58 (Supporting Information) demonstrate the metallization and dynamical stability of these two cage‐network structures of *M*C_6_ and *M*C_10_ doped by different metals at different pressures, respectively.

**Figure 2:**
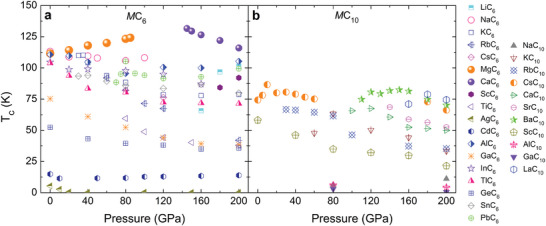
Superconducting transition temperature *T*_*c*_ dependence on pressure. (a) and (b) are corresponding to *M*C_6_ and *M*C_10_, respectively. The results are based on µ^⋆^ = 0.1.

For stable and metallic phases of *M*C_6_ and *M*C_10_, the Eliashberg electron–phonon spectral function α^2^ *F*(ω) was calculated, and then the electron–phonon coupling (EPC) constant λ, the logarithmic average of phonon frequency ω_log_, and the superconducting transition temperature *T*_*c*_ were obtained, which are summarized in Table S2 (Supporting Information). **Figure** **2** shows the dependence of *T*_*c*_ on pressure for two cage‐network carbides doped by different metals. It is found that many metal dopants lead to the superconductivity of these two cage‐network carbides, and higher *T*_*c*_ can be obtained in *M*C_6_ than *M*C_10_. Metal doping makes these two carbon structures exhibit the variety of superconductivity. Remarkably, *T*_*c*_ of *M*C_6_ is predicted to exceed 100 K, when the dopant is Li, Na, K, Mg, Ca, Al, In, Tl, and Pb. As a comparison, *T*_*c*_ of *M*C_10_ can also be above 77 K (temperature of liquid nitrogen), when the dopant is Cs, Ba, and La. With regard to pressure effect, some superconducting transition temperatures increase with the increase of pressure, some decrease with the increase of pressure, and the others first increase and then decrease with the increase of pressure. For examples, *T*_*c*_ of MgC_6_ increases with the increase of pressure from 111.6 K at ambient pressure to 124.2 K at 85 GPa, *T*_*c*_ of CaC_6_ decreases with the increase of pressure from 131.6 K at 145 GPa to 116.0 K at 200 GPa, *T*_*c*_ of CsC_10_ reaches a maximum of 86.6 K at 10 GPa. By analyzing the electronic structures and phonon characteristics, we found that the influence of pressure on superconductivity is mainly achieved by softening or hardening the phonon modes. For examples, in MgC_6_, pressurization will further soften some phonon modes, such as phonon modes near the *H* *k*‐point and in the *N* − Γ *k*‐path direction (See Figure S35, Supporting Information). On the contrary, in CaH_6_, pressurization will further harden some phonon modes, such as phonon modes in the *N* − Γ *k*‐path direction (See Figure S36, Supporting Information). However, the average pressure coefficient η = *dT*_*c*_/*dP* is relatively small. The absolute η of NaC_6_, MgC_6_, AlC_6_, GaC_6_, InC_6_, GeC_6_, CsC_10_, and ScC_10_ is respectively 0.08, 0.15, 0.07, 0.18, 0.09, 0.08, 0.10, and 0.18 KGPa^‐1^, which are lower than those of clathrate compounds such as 0.33 of CaH_6_,^[^ ^28^ ^]^ 0.37 of LaH_10_,^[^ ^33^ ^]^ 0.34 of TbH_10_,^[^ ^34^ ^]^ 0.25 of Al(BN)_3_.^[^ ^35^ ^]^ The result indicates that the high‐temperature superconductivity of cage‐network carbides produced at high pressure can be well maintained at zero pressure. The superconductivity of NaC_6_, AlC_6_, GaC_6_, and GeC_6_ can be stabilized at ambient pressure, which is different from those of Khan *et al.* ^[^ ^26^ ^]^ and Sano *et al.*,^[^ ^27^ ^]^ but supports the results of Lu *et al.* ^[^ ^25^ ^]^

**Figure 3:**
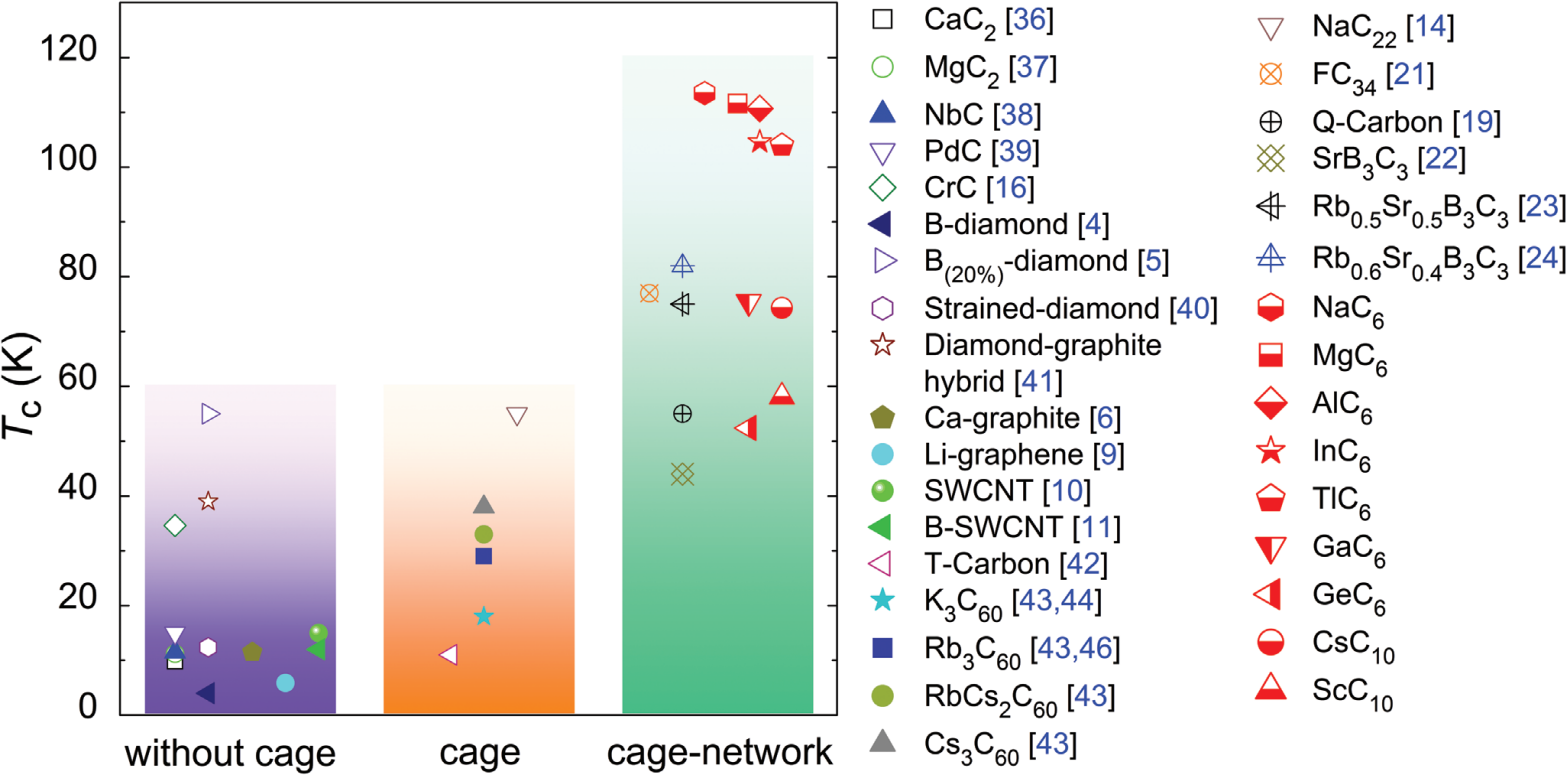
Comparison of *T*_*c*_ values of carbon‐based materials at ambient pressure. Our results are marked with the red half full symbols. The solid symbol represents the experimental results, while the hollow symbol represents the theoretical ones.

As a novel carbon structure, the superconductivity at ambient pressure is still of great interest. Figure 2 shows that NaC_6_, MgC_6_, AlC_6_, GaC_6_, InC_6_, GeC_6_, CsC_10_, and ScC_10_ can exhibit the high‐temperature superconductivity at ambient pressure. In particular, *T*_*c*_ of NaC_6_, MgC_6_, AlC_6_, InC_6_, and TlC_6_ respectively is predicted as 113.3, 111.6, 110.7, 104.6, and 104.0 K at ambient pressure. As shown in **Figure** **3**, the predicted *T*_*c*_'s of *M*C_6_ and *M*C_10_ are much >9.8 K of CaC_2_,^[^ ^36^ ^]^ 11.2 K of MgC_2_,^[^ ^37^ ^]^ 11.5 K of NbC,^[^ ^38^ ^]^ 15 K of PdC,^[^ ^39^ ^]^ and 34.6 K of CrC^[^ ^16^ ^]^ without cage structure. Importantly, the predicted *T*_*c*_ above 100 K for *M*C_6_ at ambient pressure is obviously higher than those of low dimensional carbon structures such as 4 and 55 K of boron‐doped diamond,^[^ ^4^, ^5^ ^]^ 12.4 K of strained‐diamond,^[^ ^40^ ^]^ 39 K of hybridized graphite‐diamond,^[^ ^41^ ^]^ 11.5 K of graphite‐like CaC_6_,^[^ ^6^ ^]^ 5.9 K of Li‐doped graphene,^[^ ^9^ ^]^ 15 K of SWCNT,^[^ ^10^ ^]^ and 12 K of boron‐doped SWCNT.^[^ ^11^ ^]^ It is found that our predicted *T*_*c*_ of >100 K in *M*C_6_ is also much higher than the superconducting transition temperature of those carbon structures with isolated carbon cages such as 11 K of T‐carbon,^[^ ^42^ ^]^ 18–38 K of alkali metal‐doped C_60_,^[^ ^43^, ^44^, ^45^, ^46^ ^]^ and 55 K of NaC_22_.^[^ ^14^ ^]^ This improvement in superconductivity is attributed to this novel carbon structure of cage‐network. With the same cage‐network feature, *M*C_6_ can product higher *T*_*c*_ than Q‐carbon,^[^ ^19^ ^]^ FC_34_,^[^ ^21^ ^]^ and metal‐doped (CB)_3_ clathrates.^[^ ^22^, ^23^, ^24^ ^]^

**Figure 4:**
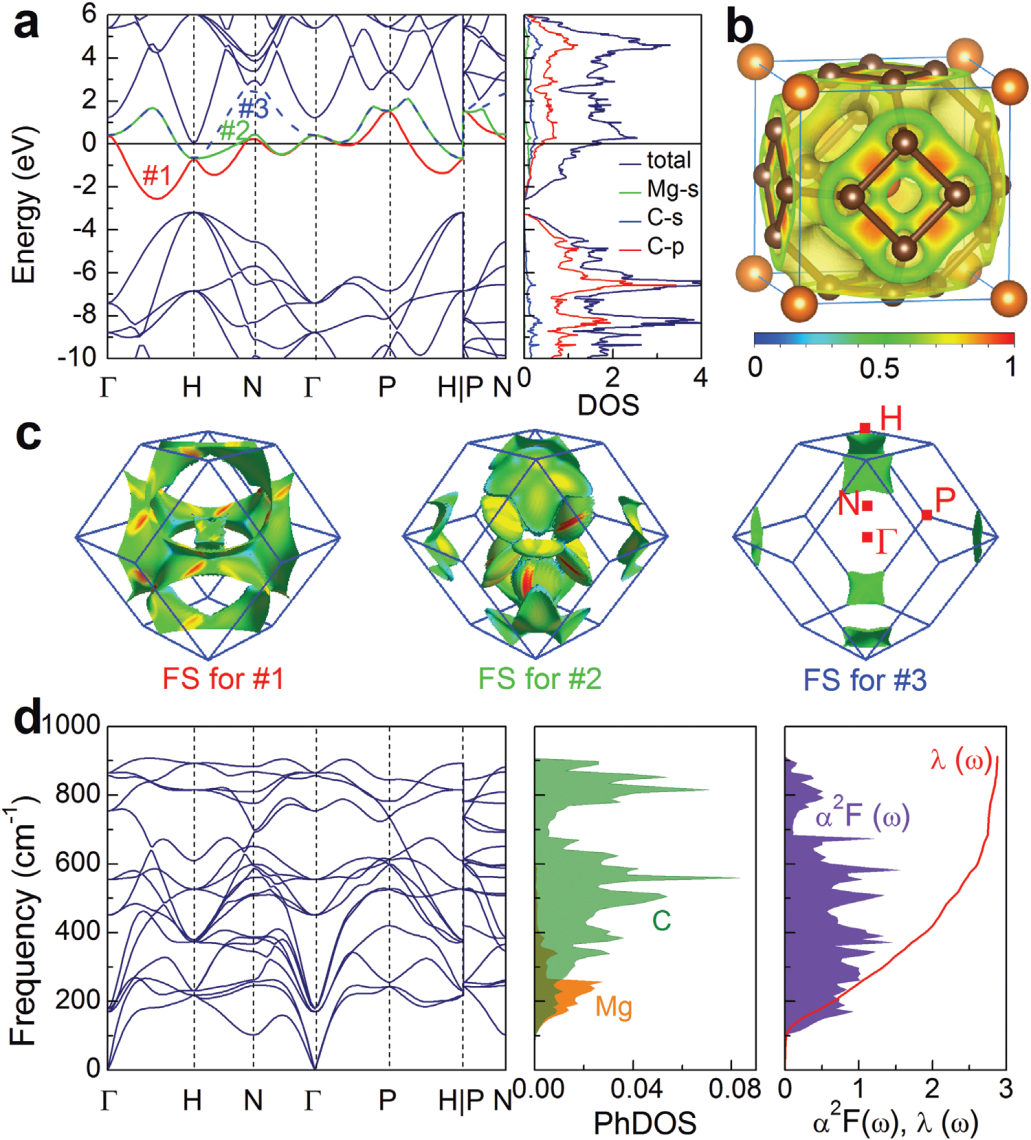
Electronic structures, phonon characteristics, and electron‐phonon coupling of MgC_6_ at 0 GPa. a) Electronic energy band structure along high‐symmetrical *k*‐point path shown in (c) and total and projected density of states (DOS) on atomic orbitals. b) Electron localization function. c) Fermi surface features in the first Brillouin zone (FBZ) corresponding to three energy bands crossing the Fermi level. d) Phonon spectra, phonon density of states (PhDOS) projected on atoms, Eliashberg spectral function α^2^ *F*(ω), and electron–phonon coupling integral λ(ω).

Pure cage‐network structure is an insulator, for example, the bandgap of C_24_‐cage‐network is about 2.5 eV,^[^ ^27^ ^]^ while the metal doping drives the transition from insulator to metal. This driven force is mainly from the charge transfer from metal to cage‐network (see Figure S59, Supporting Information). Taking MgC_6_ at 0 GPa as representative of *M*C_6_, the transferred charge from Mg to C_24_‐cage‐network is about 1.48 eMg^‐1^. The electron localization function (ELF) shown in **Figure** **4**b also displays the lack of covalent bond between Mg and C. The electrons from the metal enter the C‐2*p* orbit and are transmitted in the network of carbon cages, which causes the empty occupied conduction bands to move to the lower energy level. As a result, three energy bands cross over the Fermi level of MgC_6_, forming the complicated Fermi surface (FS) sheets including hole‐ and electron‐like and exhibiting the metallic feature as shown in Figure 4a,c. The electronic states near the Fermi level are contributed by C‐2*s* and 2*p*. The DOS value at Fermi level is 1.73 states/eV/f.u. (or 0.25 states/eV/atom) for MgC_6_. As a comparison, in the metal‐doped C_60_ with *fcc* group‐space, there are also several energy bands crossing the Fermi level, but the DOS value at Fermi level is only about 0.10 − 0.13 states/eV/atom.^[^ ^47^, ^48^ ^]^ In NaC_22_ where carbon cage is also isolated, the DOS value at Fermi level is about 0.2 states/eV/atom,^[^ ^14^ ^]^ <0.25 states/eV/atom of MgC_6_. Additionally, the cage‐network structure results in the wider energy band near Fermi level than the cage‐isolated structure, such as about 5 eV for MgC_6_ comparing with about 0.5 eV for K‐doped C_60_^[^ ^47^ ^]^ and 1.9 eV for NaC_22_.^[^ ^14^ ^]^ The high electronic DOS value at Fermi level caused by the cage‐network structure should be one of the reasons for the high‐*T*_*c*_ superconductivity of *M*C_6_ than cage‐isolated structure. Furthermore, in *M*C_6_, different metal doping leads to the difference of electronic states as shown in Figures S1– S29 (Supporting Information), which is one of the reasons why they exhibit different superconductivity.

As shown in Figures S30– S47 (Supporting Information), compared with metal‐doped C_60_, the highest phonon vibration frequency of C atoms in *M*C_6_ is greatly reduced at ambient pressure, almost from about 1600 cm^−1^ of metal‐doped C_60_^[^ ^48^ ^]^ to 900 cm^−1^ of the *M*C_6_. For example, the highest phonon frequency in MgC_6_ is 833 m^−1^ as shown in Figure 4d. However, the cage‐network structures result in the stronger EPC constants (See Table S2, Supporting Information) than that of cage‐isolated structure. As shown in Figure 4d, the EPC constant of MgC_6_ at ambient pressure is λ = 2.88, which is far >0.4 − 0.9 of metal‐doped C_60_^[^ ^48^ ^]^ and 1.12 of NaC_22_.^[^ ^14^ ^]^ Such a strong electron–phonon interaction mainly comes from the contribution of C phonon modes, and the strong EPC is a main factor for the high superconductivity of the cage‐network structure.

**Figure 5:**
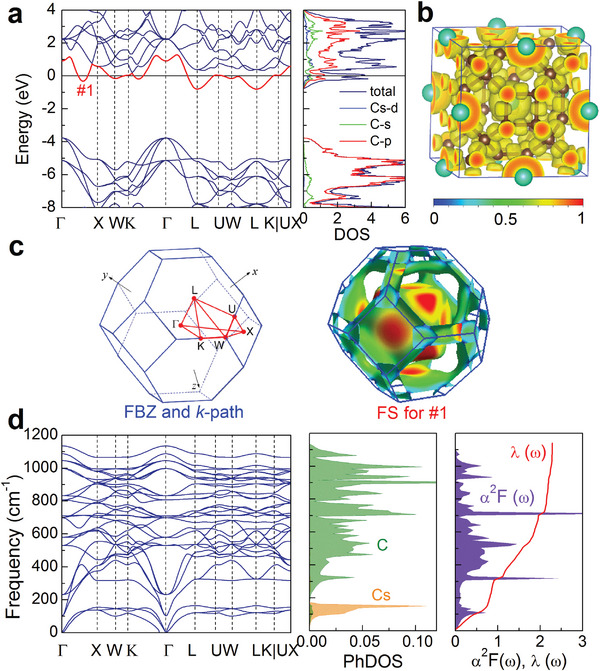
Electronic structures, phonon characteristics, and electron–phonon coupling of CsC_10_ at 0 GPa. a) Electronic energy band structure along high‐symmetrical *k*‐point path shown in (c) and total and projected density of states (DOS) on atomic orbitals. b) Electron localization function. c) Fermi surface features in the first Brillouin zone (FBZ) corresponding to one energy band crossing the Fermi level. d) Phonon spectra, phonon density of states (PhDOS) projected on atoms, Eliashberg spectral function α^2^ *F*(ω), and electron–phonon coupling integral λ(ω).

For *M*C_10_, only CsC_10_ and ScC_10_ are superconducting at ambient pressure, the *T*_*c*_ are 74.3 and 58.1 K, respectively. From the perspective of comparing electronic states and phonon structures, the reasons for the different *T*_*c*_ of *M*C_6_ and *M*C_10_ was also analyzed. In the case of CsC_10_ at ambient pressure shown in **Figure** **5**, on one hand, the transferred charge is about 0.76 eCs^‐1^, only one band crosses over the Fermi level forming hole‐ and electron‐like FS sheets. However, the DOS value at Fermi level of CsC_10_ is 0.30 states/eV/atom, slightly >0.25 states/eV/atom of MgC_6_. On the other hand, the higher phonon frequency of 1065 cm^−1^ is obtained in CsC_10_ than MgC_6_. The EPC of MgC_6_ is λ = 2.88 with ω_log_ = 449.7 K, while λ = 2.30 and ω_log_ = 346.2 K for CsC_10_. The electron–phonon interaction in CsC_10_ is slightly weaker than that in MgC_6_. Furthermore, the difference between them mainly results from the different α^2^ *F*(ω) caused by the phonon states of C atoms. As mentioned above, all the lengths of the nearest neighbor C‐C bonds are uniform in *M*C_6_, while in *M*C_10_, there are two kinds of the lengths of the nearest neighbor C‐C bonds (See Figure S60, Supporting Information). The C‐C bonding lengths forming hexagonal ring in C_32_ cage are shortened, which leads to the enhancement of some phonon vibrations, that is, the phonon becomes hard. As a result, the contribution of these phonon modes to the EPC decreases. As shown in Figures 4d and 5d, although the increase of phonon frequency increases the integration range, the phonon states (PhDOS and α^2^ *F*(ω)) in the low‐frequency region that contributes greatly to the EPC decrease significantly, which leads to the decrease of the final EPC constant.

We can see that the trend of *T*_*c*_ decreasing from *M*C_6_ to *M*C_10_ is significantly different from that of *T*_*c*_ increasing from *M*H_6_ to *M*H_9_ and then to *M*H_10_. From the perspective of electronic structures and phonon spectra characteristics, we have analyzed their differences in detail. For the doping of the same element at the same pressure (See Figures S1– S29, Supporting Information), the DOS value at Fermi level increases from *M*C_6_ to *M*C_10_, which is similar to that of from YH_6_ to YH_9_ and then to YH_10_.^[^ ^18^ ^]^ At the same pressure, the highest frequency of phonon vibration increases with the increase of C content from *M*C_6_ to *M*C_10_, and this phenomenon of the highest phonon frequency increasing with the increase of H content was also observed in hydrides such as from LaH_8_ to LaH_10_.^[^ ^17^ ^]^ However, the *T*_*c*_ of carbides and hydrides exhibit opposite trends, with the former decreasing with an increase in C content and the latter increasing with an increase in H content. We analyzed the phonon spectrum and found that this reason may mainly come from the decrease of soft phonon modes with the increase of C content in carbides, and the increase of soft phonon modes with the increase of H content in hydrides.^[^ ^17^, ^18^ ^]^ The stronger hybridization between carbon atoms (See Figure S61, Supporting Information) increases their stability at ambient pressure and also makes phonons harder, resulting in a decrease in superconductivity.

**Figure 6:**
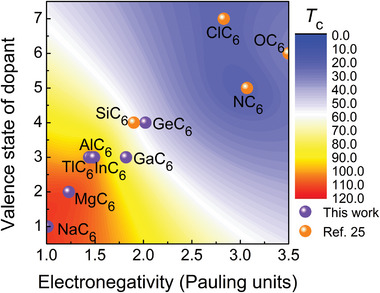
Phase diagram of *T*_*c*_ and dopants. The dependence of *T*_*c*_ on the electronegativity and valence states of dopant for *M*C_6_ superconductor at ambient pressure. The contour colors imply the range of *T*_*c*_ values. Results of non‐metal doping are taken from previous reports.^[^ ^25^ ^]^

Although the doped metal only provides the transferred charge and only contributes about a quarter to the total EPC constant, the different doped metals also cause the diversity of superconductivity. By analyzing the relationship between different metals and transition temperature, we found some simple rules. Focusing on those superconductors at ambient pressure combining with previous studies,^[^ ^25^ ^]^ as shown in **Figure** **6**, it is found that the *T*_*c*_ decreases with the increase of Allred‐Rochow electronegativity^[^ ^49^ ^]^ and valence state of *M* in *M*C_6_. Alkali metals and alkaline earth metals exhibit better advantages in exploring the higher *T*_*c*_ in cage‐network carbides, which is just why these metals were mainly used in doping C_60_^[^ ^12^, ^13^ ^]^ or other carbides.^[^ ^14^, ^31^, ^32^ ^]^ As a result, Figure 6 suggests the direction of exploring high‐*T*_*c*_ clathrate carbide superconductors, toward to metals with low electronegativity and low valence state. This law also seems to be applicable to hydrides with clathrate structure formed by H atoms. High or near room‐temperature superconductivity is often observed in clathrate hydrides‐doped by metal with low electronegativity and valence state. The superconductivity with *T*_*c*_'s over 200 K was predicted theoretically in CaH_6_,^[^ ^28^ ^]^ YH_6_,^[^ ^50^ ^]^ MgH_6_,^[^ ^51^ ^]^ LaH_10_,^[^ ^17^ ^]^ YH_10_,^[^ ^17^, ^18^ ^]^ ThH_10_,^[^ ^52^ ^]^ AcH_10_,^[^ ^53^ ^]^ TbH_10_,^[^ ^54^ ^]^ CaYH_12_,^[^ ^55^ ^]^ and Li_2_MgH_16_,^[^ ^56^ ^]^ respectively. Metals such as La, Y, Li, Mg, and Ca have relatively weak electronegativity, and their doping with hydrides leads to higher *T*_*c*_. In addition, we have investigated the influence of dopant content on *T*_*c*_. Changing the doping concentration, the *T*_*c*_ of *M*C_6_ which can be stable at ambient was calculated. As listed in Table S3 (Supporting Information), the metal content in *M*C_6_ is reduced, and *T*_*c*_ is significantly reduced. The result shows that the superconducting transition temperature is sensitive to the metal doping concentration, which means that we can obtain higher *T*_*c*_ by adjusting the metal content. For defect effects, Figures S62 and S63 (Supporting Information) show the defect structures of C atomic vacancy and doping charge and phonon spectra with these defects, taking NaC_6_ and CsC_10_ at 0 GPa as examples, respectively. The results indicate that the systems are dynamically unstable under defects. Hence, we speculate that attempting to regulate superconductivity by adjusting defects in *M*C_6_ and *M*C_10_, such as C vacancies and doping charges, is not a very effective means.

Finally, the mechanical and thermodynamic stabilities and the possibility of experimental synthesis of these two kinds of cage‐network carbides were simply analyzed. The mechanical stability was analyzed by calculating the elastic constants of *M*C_6_ and *M*C_10_, especially for systems that can superconduct at ambient pressure (See Table S4, Supporting Information). The results show that both *M*C_6_ and *M*C_10_ meet the criteria of elastic stability, which means that these cage‐network structures are mechanically stable. The enthalpy of formation were calculated within the quasi‐harmonic approximations (QHA)^[^ ^57^ ^]^ when the possibly synthesizing routes of metal + diamond and metal + graphite were assumed. Considering the pressure range of 0 − 200 GPa and the temperature range of 0 − 2000 K (Figures S64 and S65, Supporting Information), it is found that the enthalpy of formation is negative. This means that these two kinds of cage‐network structures are thermodynamically stable and can be synthesized starting from metal and diamond or graphite under certain conditions. The order of magnitude of decomposition enthalpy is in the range of −0.45 − −0.1 eV for *M*C_6_ and *M*C_10_. Clathrate hydrides generally exist under higher pressure. Compared with the successfully synthesized hydrides such as LaH_10_,^[^ ^1^, ^2^ ^]^ YH_6_,^[^ ^58^, ^59^ ^]^ and CaH_6_,^[^ ^60^ ^]^ it was found that the decomposition enthalpy is comparable. For example, the decomposition enthalpies are about ‐0.35 eV at 150 GPa and 0.40 eV at 300 GPa for LaH_10_,^[^ ^17^ ^]^ ‐0.72 eV at 160 GPa for YH_6_,^[^ ^50^ ^]^ and ‐0.9 eV at 150 GPa for CaH_6_,^[^ ^28^ ^]^ respectively. This implies the possibility of experimental synthesis of clathrate carbides at a certain temperature and pressure. More synthesis routines have been explored by comparing the enthalpy of formation between *M*C_6_ (or *M*C_10_) and metal + C_24_ cage (or C_32_ cage), as well comparing the enthalpy of formation of metal carbide + carbon substance (such as graphite or diamond). The results (See Figure S66, Supporting Information) not only show that *M*C_6_ and *M*C_10_ have good thermodynamic stability, but also provides some possible synthesis routines. For example, MgC_6_ can be synthesized through various forms such as Mg + carbon substance (such as graphite or diamond), MgC + carbon substance, MgC_2_ + carbon substance, *etc*. Actually, some cage‐isolated systems and cage‐coupled materials have been reported to be successfully experimentally synthesized, such as SiB_6_,^[^ ^61^ ^]^ metal‐doped C_60_ superconductors,^[^ ^12^, ^13^, ^62^ ^]^ and carbon–boron clathrate structure.^[^ ^63^ ^]^ Zhu et al. suggested a synthetic route of carbon–boron clathrates that metal carbides and borides were mixed and heated to 2500 K and the pressure was controlled in the range of 50 − 150 GPa.^[^ ^63^ ^]^ As a reference, clathrate carbides can be synthesized under the similar conditions, especially after removing pressure, these carbides will maintain good stability and superconductivity, as the pressure effect mentioned above. Moreover, the quasi‐hexagonal‐phase fullerene has been successfully synthesized, where the C_60_ cage‐network was formed via an interlayer bonding.^[^ ^64^ ^]^ These experiments also provide a reference for the synthesis of these carbon‐cage‐network structures.

## 3 Conclusion 

In summary, based on C_24_ and C_32_ cages, we have respectively designed two carbon‐cage‐network structures and systematically studied their structural stability, electronic properties, phonon spectra, and electron–phonon interactions after doping metals. The strong electron–phonon interactions were obtained in these two kinds of carbon‐cage‐network. The predicted *T*_*c*_s in *M*C_10_ are slightly lower than those in *M*C_6_. At ambient pressure, the highest *T*_*c*_ induced by C_32_‐cage‐network structure is 74 K that is obtained in CsC_10_. Remarkably, C_24_‐cage‐network structure‐doped by Na, Mg, Al, In, and Tl exhibits the superconductivity above 100 K at ambient pressure, which is far higher than those in graphite, fullerene, and other carbides. The results indicate that the superconductivity of cage‐network carbides is sensitive to the electronegativity and concentration of dopant. Our current results provide a useful route for designing high‐*T*_*c*_ superconductors. And we expect that the work will stimulate further experimental and theoretical studies for exploration of carbide high‐temperature superconductors.

## 4 Simulation Details

All calculations of structural optimization and electronic structures were carried out by using the density functional theory of Perdew–Burke–Ernzerhof (PBE) generalized gradient approximation (GGA)^[^ ^65^ ^]^ and the projector augmented wave pseudopotential^[^ ^66^ ^]^ as implemented in the Vienna ab initio simulation package (VASP).^[^ ^67^, ^68^ ^]^ The plane wave cut‐off energy was set as 600 eV. The *k*‐point of the Brillouin zone was 0.03 Å^−1^ interval distribution of Monkhorst‐Pack for the optimization of structures, and the *k*‐point interval of the total energy self‐consistent calculation was 0.02 Å^−1^ or better. Convergence thresholds were set as 10^−5^ eV in energy and 10^−3^ eVÅ^‐1^ in force.

Within the framework of density functional perturbation theory and PBE‐GGA functional, QUANTUM ESPRESSO package (QE)^[^ ^69^, ^70^ ^]^ was used to calculate the phonon frequency (ω) and the Eliashberg electron–phonon spectral function [α^2^ *F*(ω)]. Based on α^2^ *F*(ω), the electron–phonon coupling constant (λ, EPC) of these clathrate compounds was calculated, which is defined by integration over the entire frequency domain of α^2^ *F*(ω):

(1) $$\lambda =2\int_{0}^{\infty }\frac{\alpha^{2}F\left(\omega \right)}{\omega }d\omega .$$

Then *T*_c_ was calculated by Allen‐Dynes‐corrected McMillan equation^[^ ^71^ ^]^:

(2) $$T_{c}=f_{1}f_{2}\frac{\omega_{\log}}{1.2}\exp\left[-\frac{1.04(1+\lambda )}{\lambda -\mu^{*}\left(1+0.62\lambda \right)}\right],$$

where, the factor *f*_1_ *f*_2_ is decided by the λ, µ^⋆^, ω_log_, and mean square frequency ($\bar{\omega_{2}}$),^[^ ^71^ ^]^ and the logarithmic average of phonon frequency ω_log_ is written as:

(3) $$\omega_{\log}=\exp\left[\frac{2}{\lambda }\int_{0}^{\infty }\frac{\alpha^{2}F\left(\omega \right)\log\left(\omega \right)}{\omega }d\omega )\right].$$

The typical value of Coulomb pseudopotential µ^⋆^ was set as 0.1 for all clathrate carbides. Ultrasoft pseudopotentials for metal and carbon were employed in this calculation. And a *k*‐mesh of 16 × 16 × 16 in the first Brillouin zone was used in the calculation of the electron–phonon interaction matrix element and a *q*‐mesh of 4 × 4 × 4 was used for the phonon spectra calculation. The cut‐off energies for wave function and charge density were set as 80 Ry and 600 eV, respectively. At the same time, the forces and stresses of the convergent structure were optimized and controlled within the error range of VASP and QE programs.

## Conflict of Interest

The authors declare no conflict of interest.

## Supporting information

Supporting Information

## Data Availability

The data that support the findings of this study are available from the corresponding author upon reasonable request.
