# Supplementary material for: Superconductivity Above 100 K Predicted in Carbon‐Cage Network
Source: Adv Sci (Weinh). 2023 Oct 9;10(33):2303639. doi: 10.1002/advs.202303639 (PMC10667821; doi:10.1002/advs.202303639)
Supplement: Supplementary file 1 — Supporting Information [file ADVS-10-2303639-s001.pdf]

ADVANCED SCIENCE
Open Access

Supporting Information

for *Adv. Sci.*, DOI 10.1002/advs.202303639

Superconductivity Above 100 K Predicted in Carbon-Cage Network

Yu-Long Hai, Meng-Jing Jiang, Hui-Li Tian, Guo-Hua Zhong, Wen-Jie Li, Chun-Lei Yang, Xiao-Jia Chen and Hai-Qing Lin*

Supplementary information

Superconductivity above 100 K predicted in carbon-cage network

Yu-Long Hai,[1, 2] Meng-Jing Jiang,[1, 2] Hui-Li Tian,[1, 2] Guo-Hua Zhong,[1, 3, *] Wen-Jie Li, [1, 3] Chun-Lei Yang, [1, 3, †] Xiao-Jia Chen,[4, 5, ‡] and Hai-Qing Lin[6, §]

[1]*Shenzhen Institute of Advanced Technology, Chinese Academy of Sciences, Shenzhen 518055, China*

[2]*Nano Science and Technology Institute, University of Science and Technology of China, Suzhou 215123, China*

[3]*University of Chinese Academy of Sciences, Beijing 100049, China*

[4]*School of Science, Harbin Institute of Technology, Shenzhen 518055, China*

[5]*Center for High Pressure Science and Technology Advanced Research, Shanghai 201203, China*

[6]*School of Physics, Zhejiang University, Hangzhou 310027, China*

Outline

1. Metallization and dynamically stability statistics 3

2. Calculated total density of states (DOS) for $M\mathrm{C}_x$ (x = 6 and 10) at different pressures 4

3. Calculated phonon spectra of $M\mathrm{C}_x$ (x=6 and 10) at different pressures 18

4. Electron-phonon coupling constant λ, logarithmic average of phonon frequency $\omega_{\log}$ (K), and transition temperature T_c (K) 33

5. Charge transfer 37

6. The bonding length of the nearest neighbor C-C 37

7. 2D ELF comparison between hydrides and carbides 38

8. Influence of dopant concentration on T_c for C_{24}-cage-network structures 38

9. Defect effect of NaC_6 and CsC_{10} at 0 GPa 39

10. Mechanical stability: elastic constants 40

11. Thermodynamic stability: enthalpy of formation 41

12. The size dependence of the calculation 43

13. POSCAR files of $\boldsymbol{Im3m}$ – $M\mathrm{C}_6$ 44

14. PO POSCAR files of $\boldsymbol{Fm3m}$ - $M\mathrm{C}_{10}$ with the highest T_c 46

1. Metallization and dynamically stability statistics

Table S1 System satisfying both metallization and stability conditions is marked with √, otherwise it is marked with ×. The stable pressure range is shown in brackets.

M	MC_6	MC_{10}
Li	√ (160-200 GPa)	×
Na	√ (0-100 GPa)	√ (80-200 GPa)
K	√ (30-200 GPa)	√ (60-200 GPa)
Rb	√ (60-200 GPa)	√ (30-200 GPa)
Cs	√ (80-120 GPa)	√ (0-60, 180-200 GPa)
Be	×	×
Mg	√ (0-85 GPa)	×
Ca	√ (145-200 GPa)	√ (100~200 GPa)
Sr	×	√ (140-200 GPa)
Ba	×	√ (110-200 GPa)
Sc	√ (180-200 GPa)	√ (0-200 GPa)
Y	×	×
Ti	√ (80-200 GPa)	×
Zr	×	×
Hf	×	×
Cu	×	×
Ag	√ (0-200 GPa)	×
Zn	×	×
Cd	√ (0-200 GPa)	×
Al	√ (0-200 GPa)	√ (80-200 GPa)
Ga	√ (0-200 GPa)	√ (80-200 GPa)
In	√ (0-160 GPa)	√ (80-200 GPa)
Tl	√ (0-200 GPa)	√ (80-200 GPa)
Ge	√ (0-200 GPa)	√ (160-200 GPa)
Sn	√ (30-200 GPa)	√ (160-200 GPa)
Pb	√ (70-200 GPa)	√ (160-200 GPa)
La	×	√ (160-200 GPa)

2. Calculated total density of states (DOS) for MC_x (x = 6 and 10) at different pressures

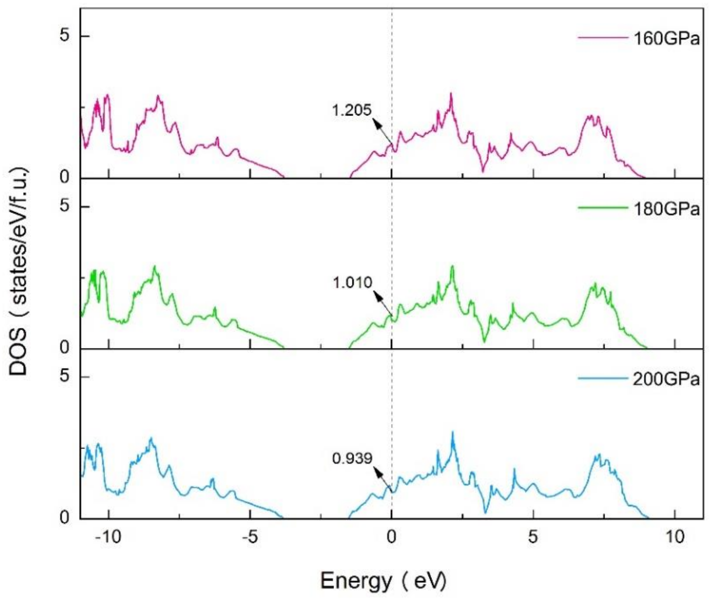

Fig. S1 Calculated total DOS of LiC_6 at different pressures. Zero energy denotes the Fermi level. The DOS value at Fermi level is present.

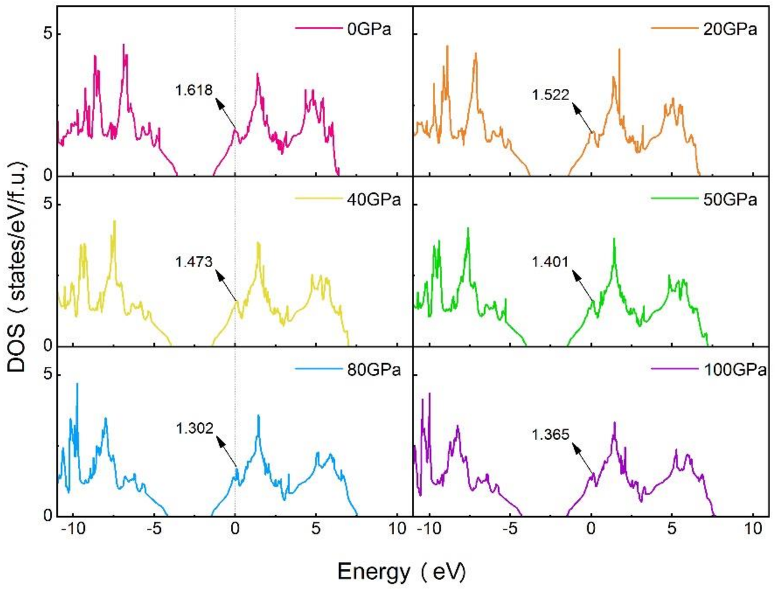

Fig. S2 Calculated total DOS of NaC_6 at different pressures. Zero energy denotes the Fermi level. The DOS value at Fermi level is present.

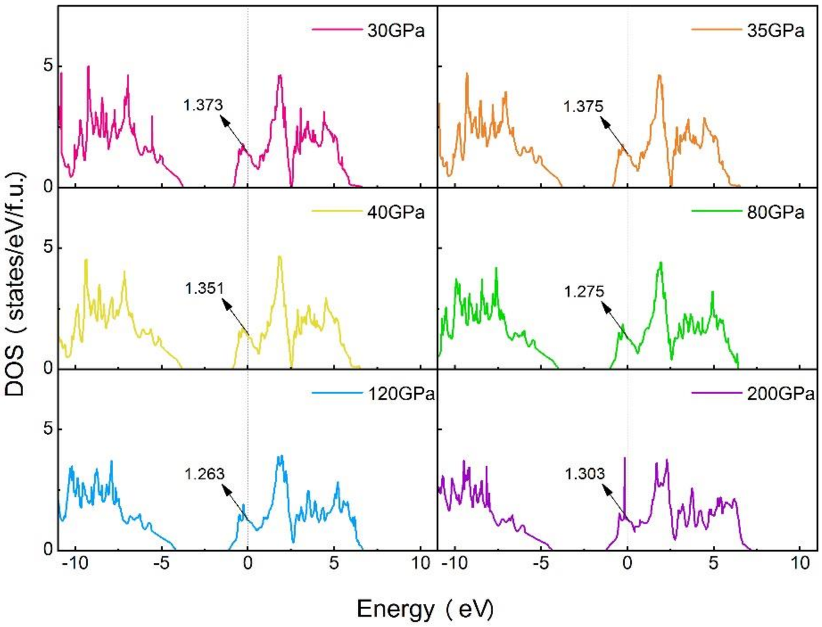

Fig. S3 Calculated total DOS of KC_6 at different pressures. Zero energy denotes the Fermi level. The DOS value at Fermi level is present.

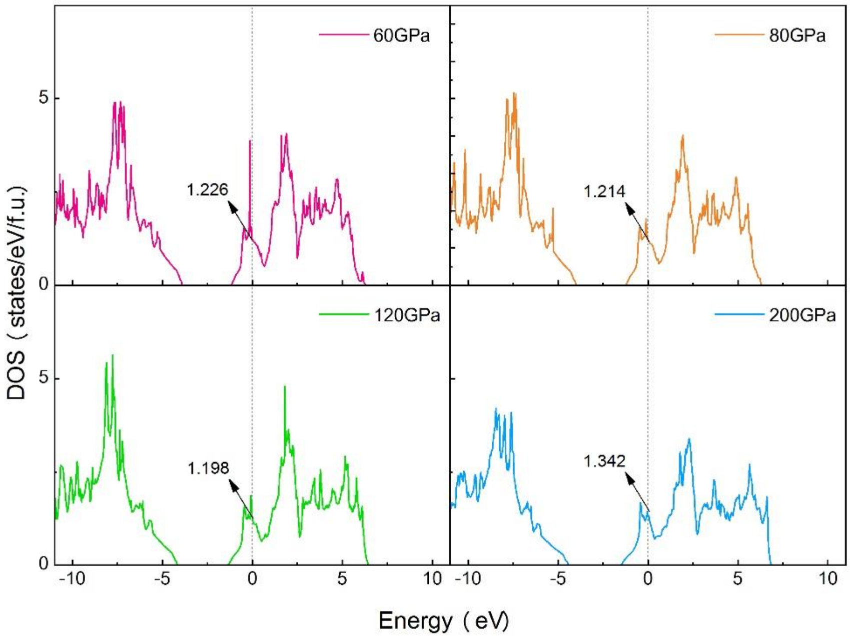

Fig. S4 Calculated total DOS of RbC_6 at different pressures. Zero energy denotes the Fermi level. The DOS value at Fermi level is present.

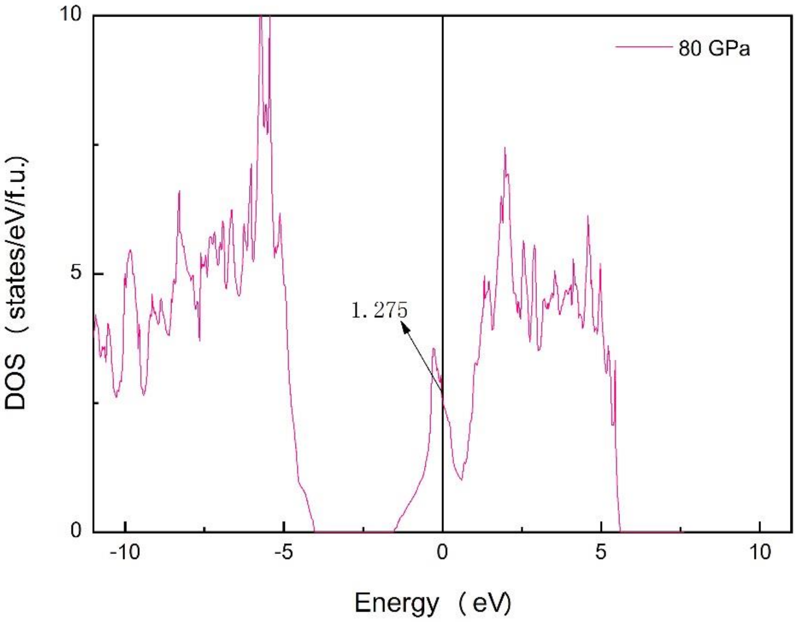

Fig. S5 Calculated total DOS of CsC_6 at different pressures. Zero energy denotes the Fermi level. The DOS value at Fermi level is present.

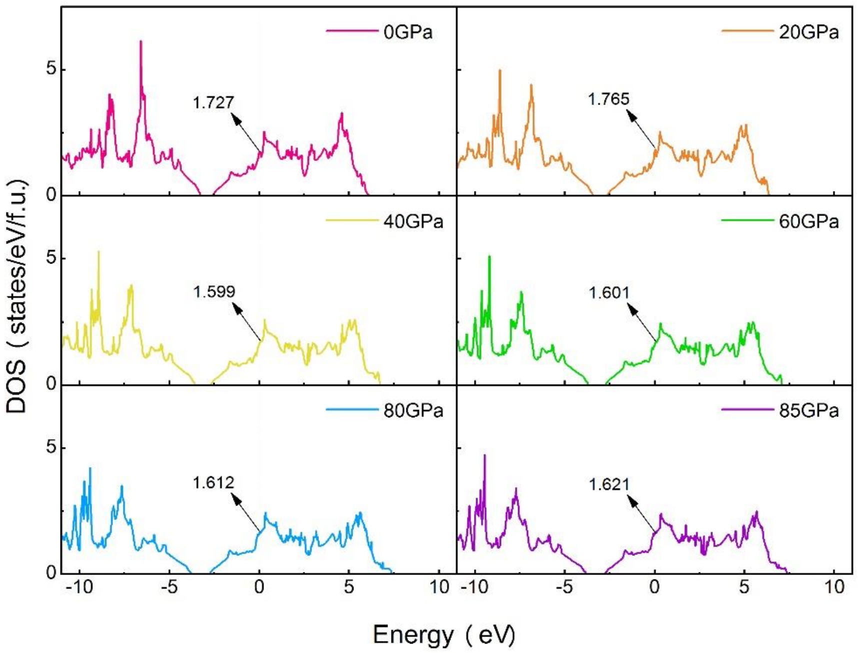

Fig. S6 Calculated total DOS of MgC_6 at different pressures. Zero energy denotes the Fermi level. The DOS value at Fermi level is present.

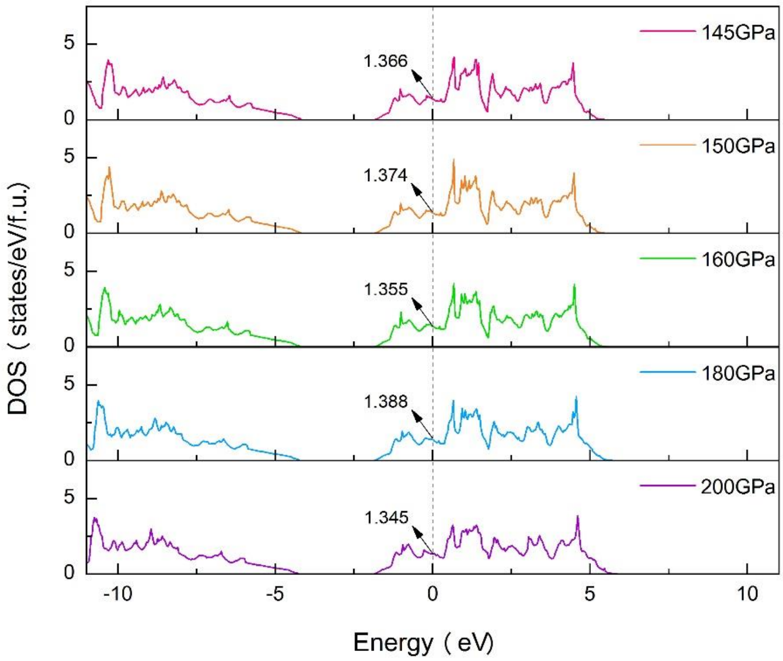

Fig. S7 Calculated total DOS of CaC_6 at different pressures. Zero energy denotes the Fermi level. The DOS value at Fermi level is present.

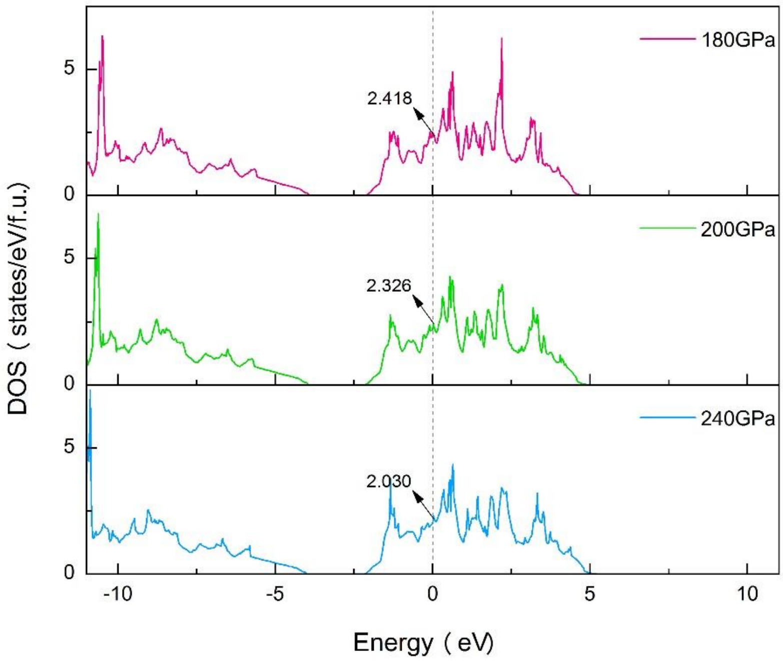

Fig. S8 Calculated total DOS of ScC_6 at different pressures. Zero energy denotes the Fermi level. The DOS value at Fermi level is present.

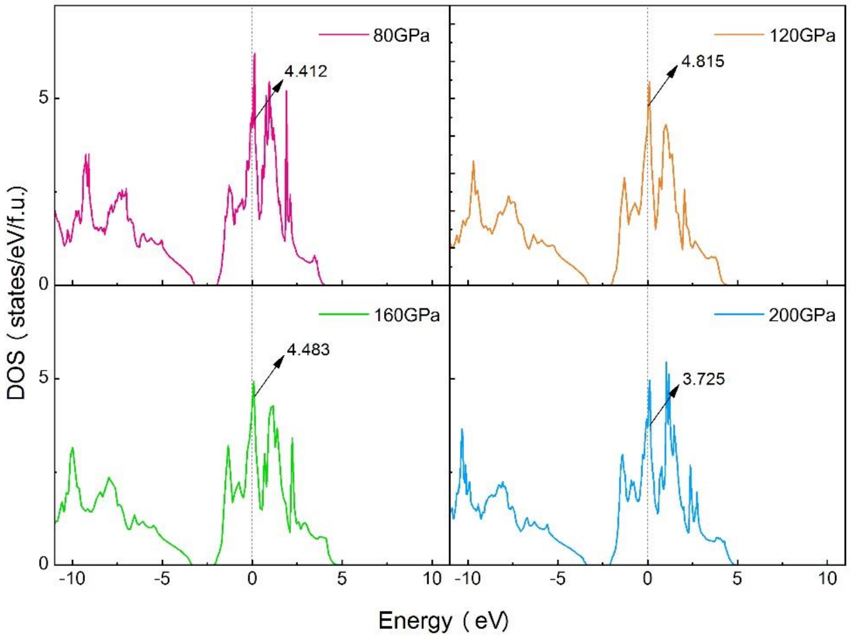

Fig. S9 Calculated total DOS of TiC_6 at different pressures. Zero energy denotes the Fermi level. The DOS value at Fermi level is present.

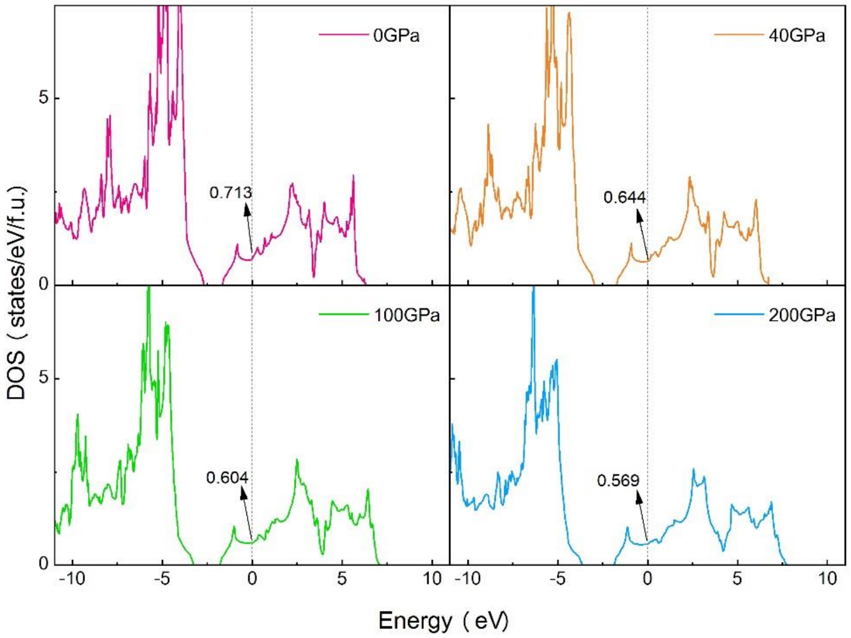

Fig. S10 Calculated total DOS of AgC_6 at different pressures. Zero energy denotes the Fermi level. The DOS value at Fermi level is present.

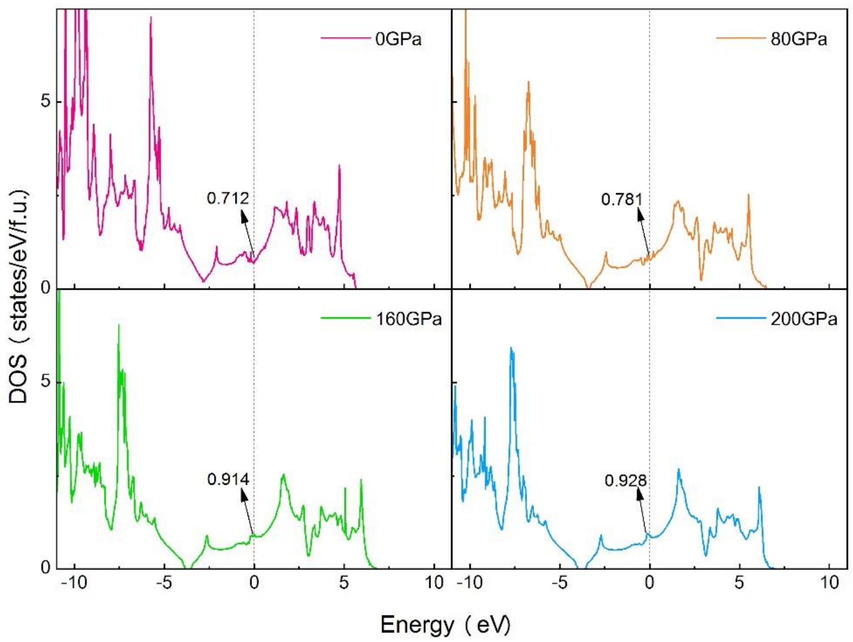

Fig. S11 Calculated total DOS of CdC_6 at different pressures. Zero energy denotes the Fermi level. The DOS value at Fermi level is present.

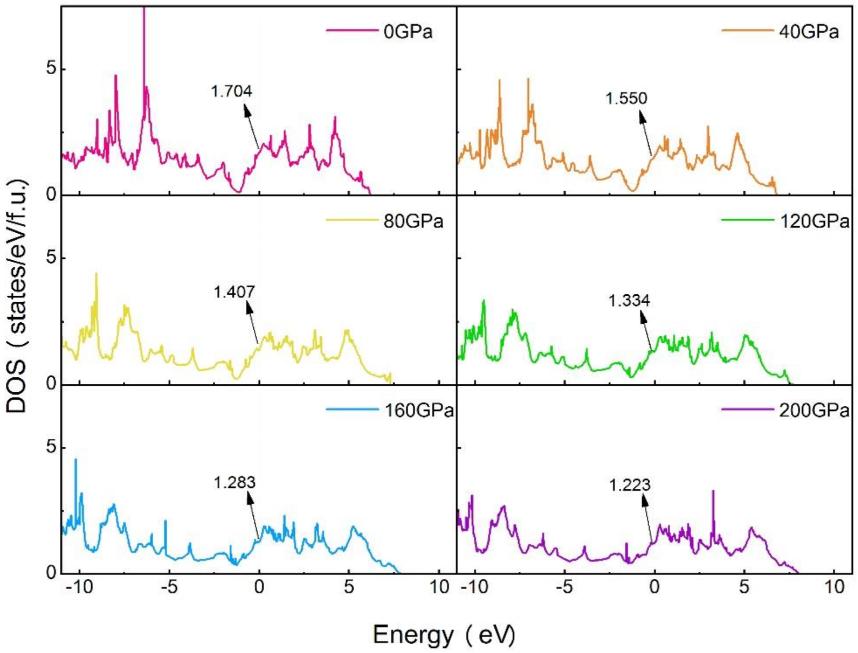

Fig. S12 Calculated total DOS of AlC_6 at different pressures. Zero energy denotes the Fermi level. The DOS value at Fermi level is present.

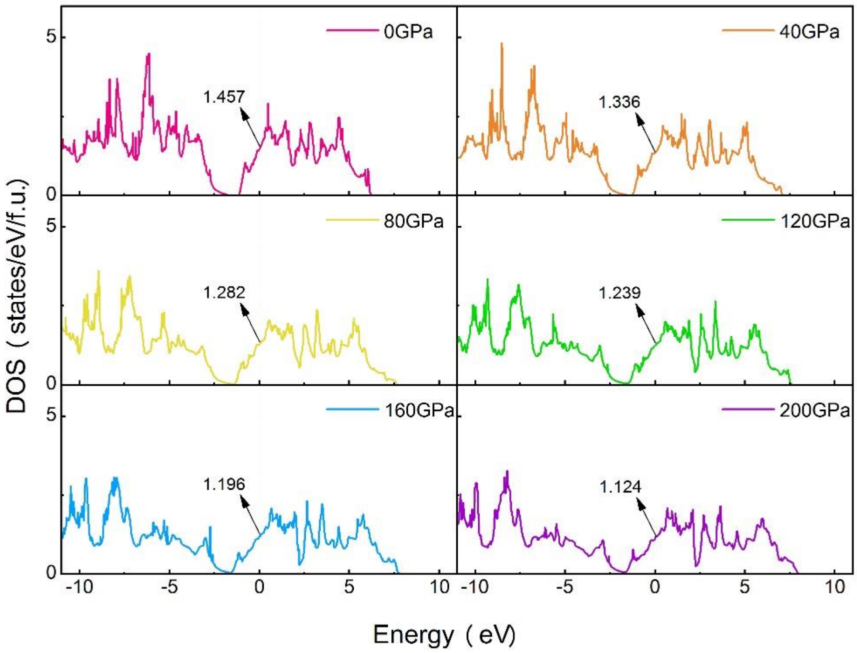

Fig. S13 Calculated total DOS of GaC_6 at different pressures. Zero energy denotes the Fermi level. The DOS value at Fermi level is present.

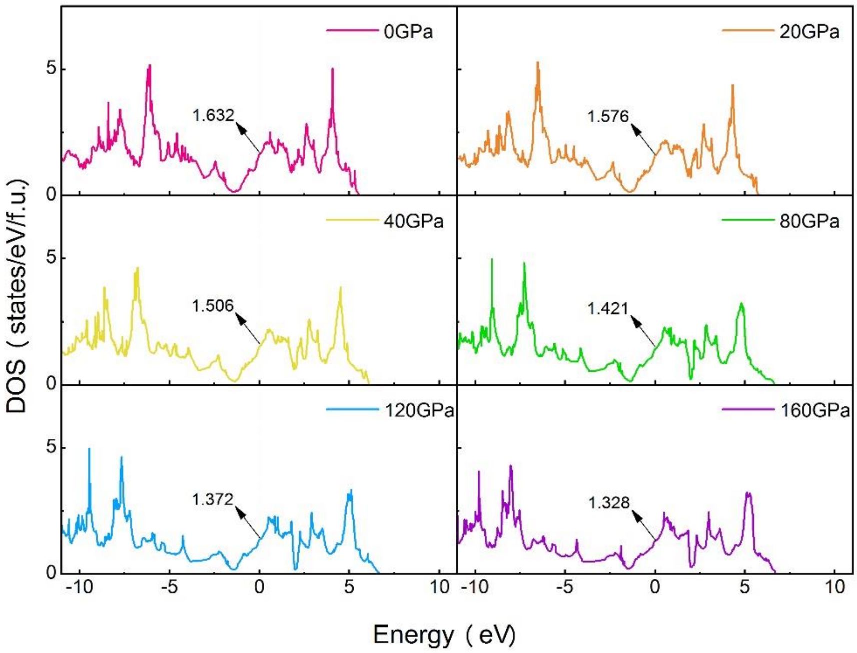

Fig. S14 Calculated total DOS of InC_6 at different pressures. Zero energy denotes the Fermi level. The DOS value at Fermi level is present.

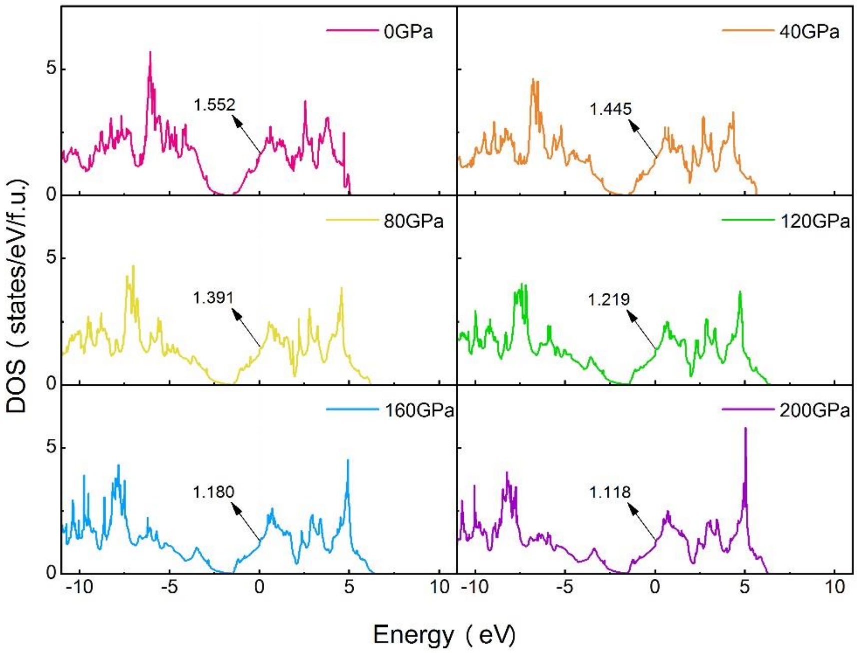

Fig. S15 Calculated total DOS of TlC_6 at different pressures. Zero energy denotes the Fermi level. The DOS value at Fermi level is present.

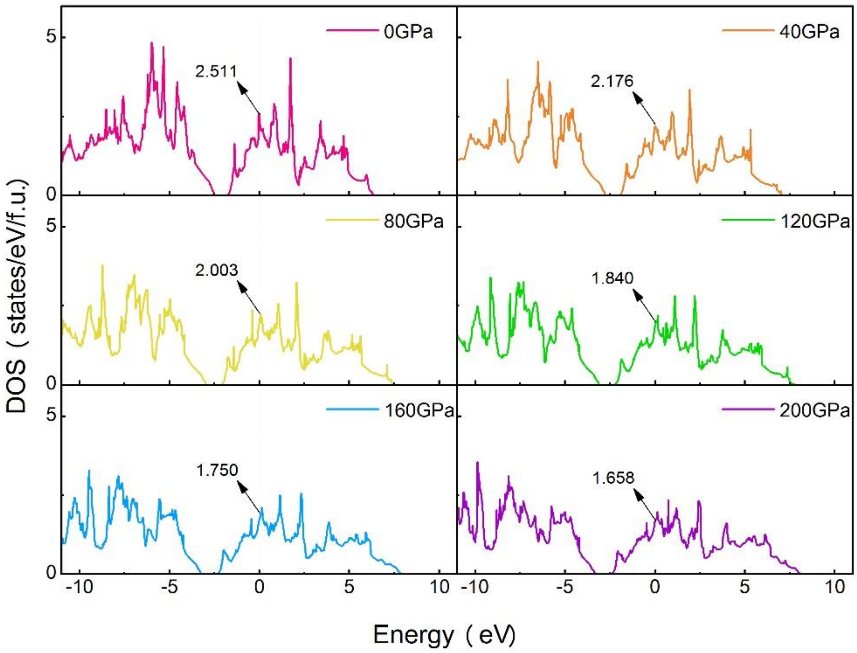

Fig. S16 Calculated total DOS of GeC_6 at different pressures. Zero energy denotes the Fermi level. The DOS value at Fermi level is present.

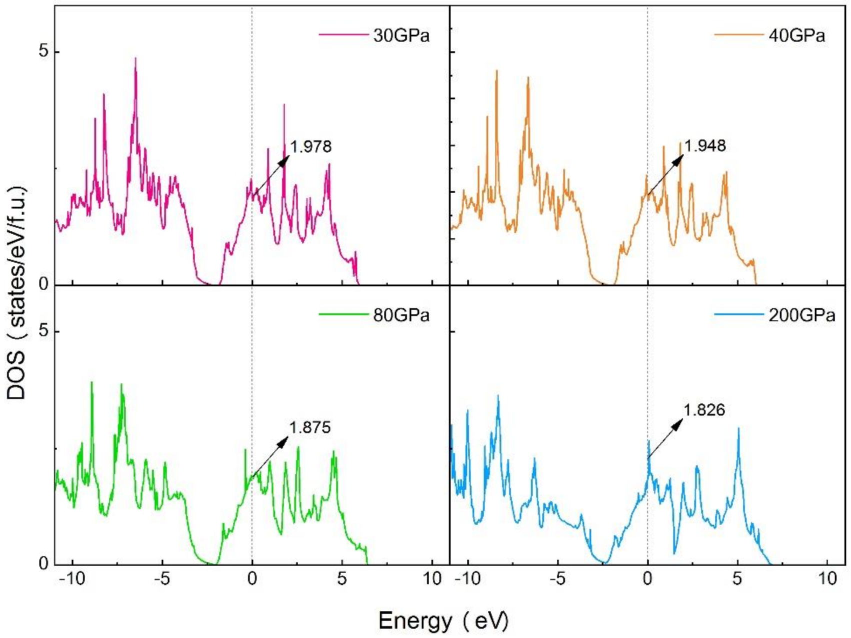

Fig. S17 Calculated total DOS of SnC_6 at different pressures. Zero energy denotes the Fermi level. The DOS value at Fermi level is present.

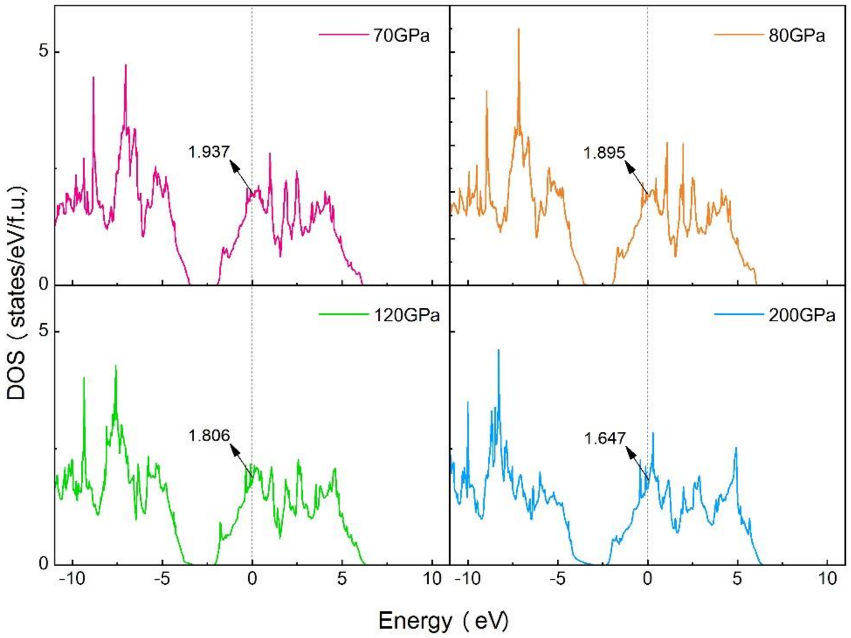

Fig. S18 Calculated total DOS of PbC_6 at different pressures. Zero energy denotes the Fermi level. The DOS value at Fermi level is present.

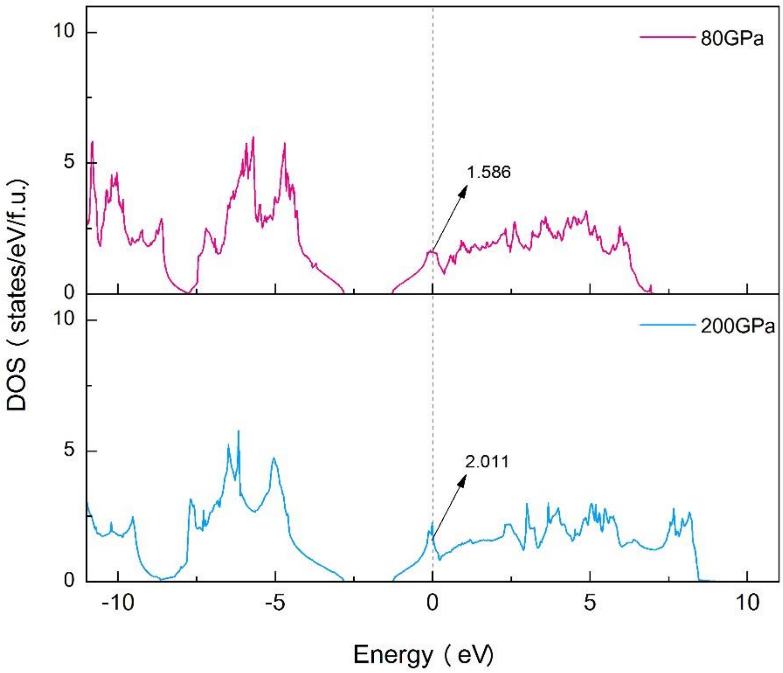

Fig. S19 Calculated total DOS of NaC_{10} at different pressures. Zero energy denotes the Fermi level. The DOS value at Fermi level is present.

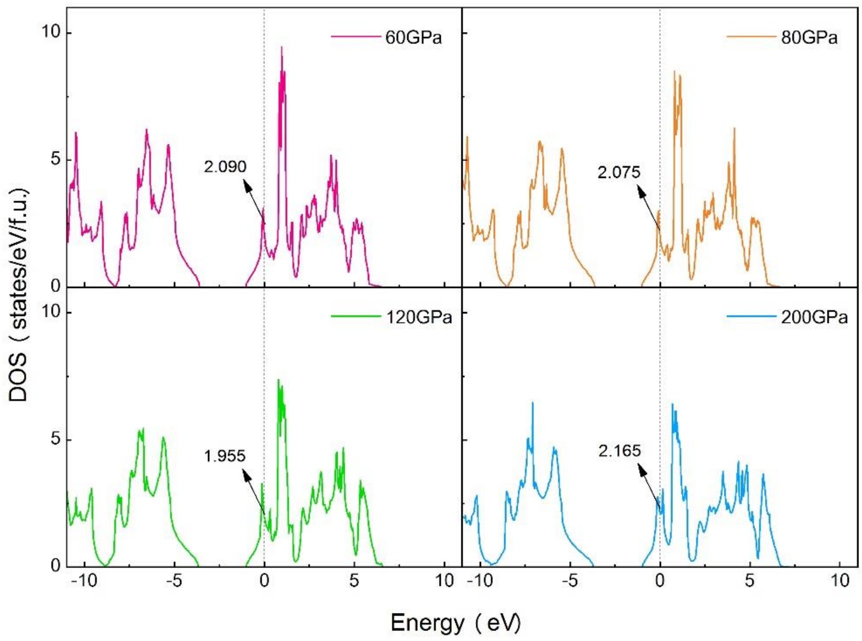

Fig. S20 Calculated total DOS of KC_{10} at different pressures. Zero energy denotes the Fermi level. The DOS value at Fermi level is present.

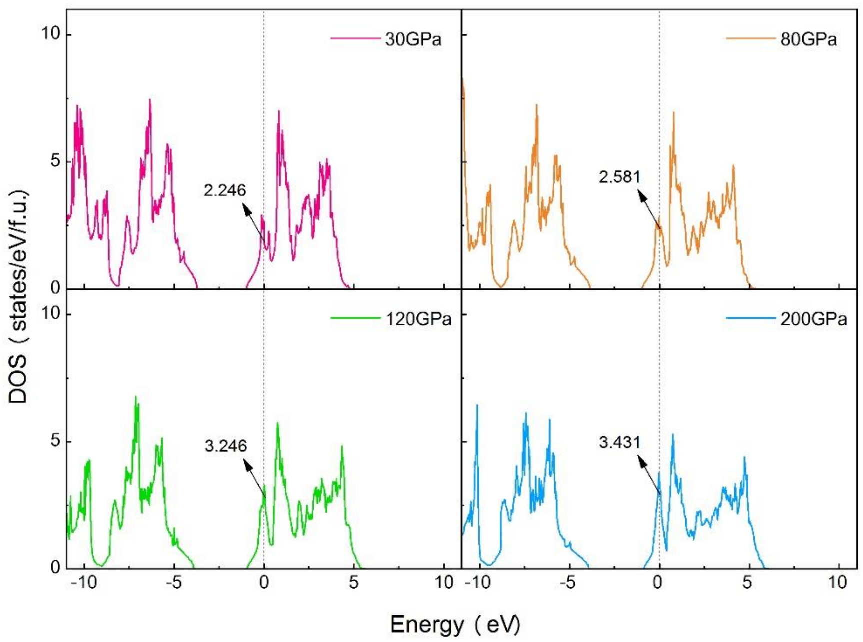

Fig. S21 Calculated total DOS of RbC_{10} at different pressures. Zero energy denotes the Fermi level. The DOS value at Fermi level is present.

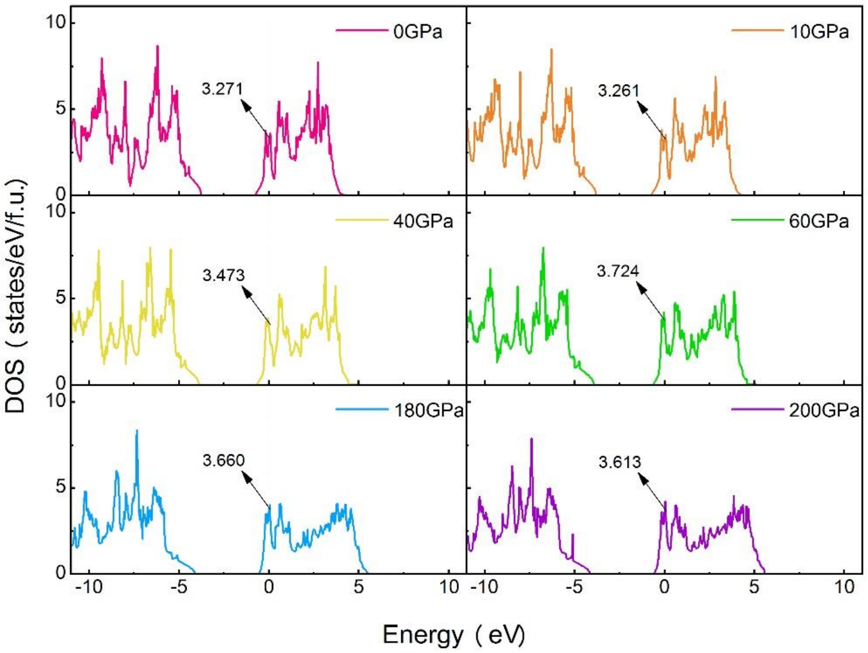

Fig. S22 Calculated total DOS of CsC_{10} at different pressures. Zero energy denotes the Fermi level. The DOS value at Fermi level is present.

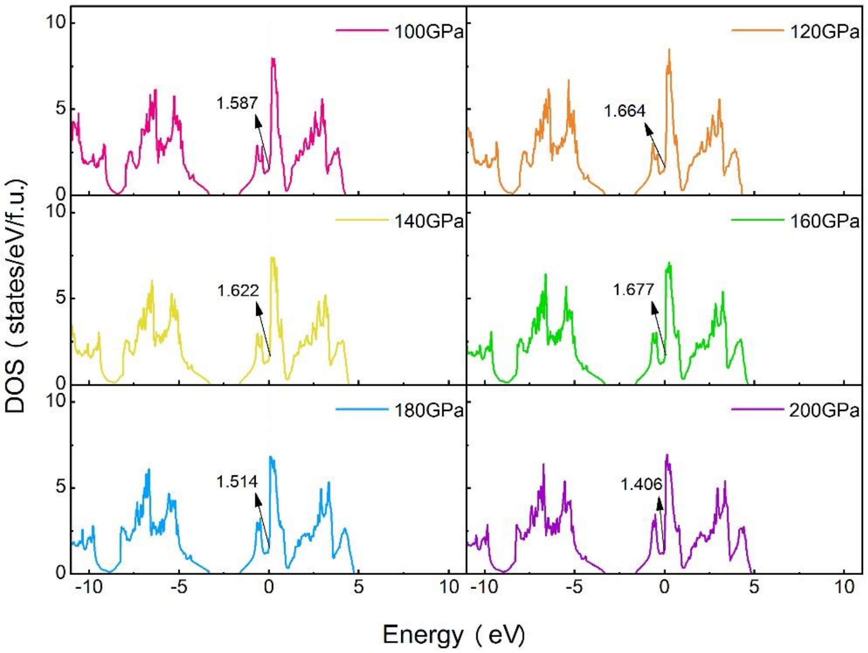

Fig. S23 Calculated total DOS of CaC_{10} at different pressures. Zero energy denotes the Fermi level. The DOS value at Fermi level is present.

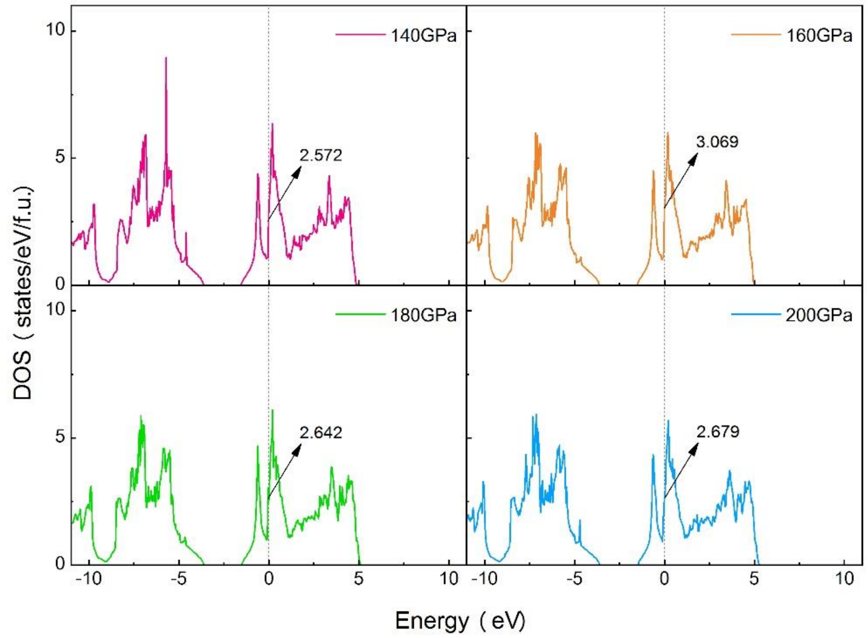

Fig. S24 Calculated total DOS of SrC_{10} at different pressures. Zero energy denotes the Fermi level. The DOS value at Fermi level is present.

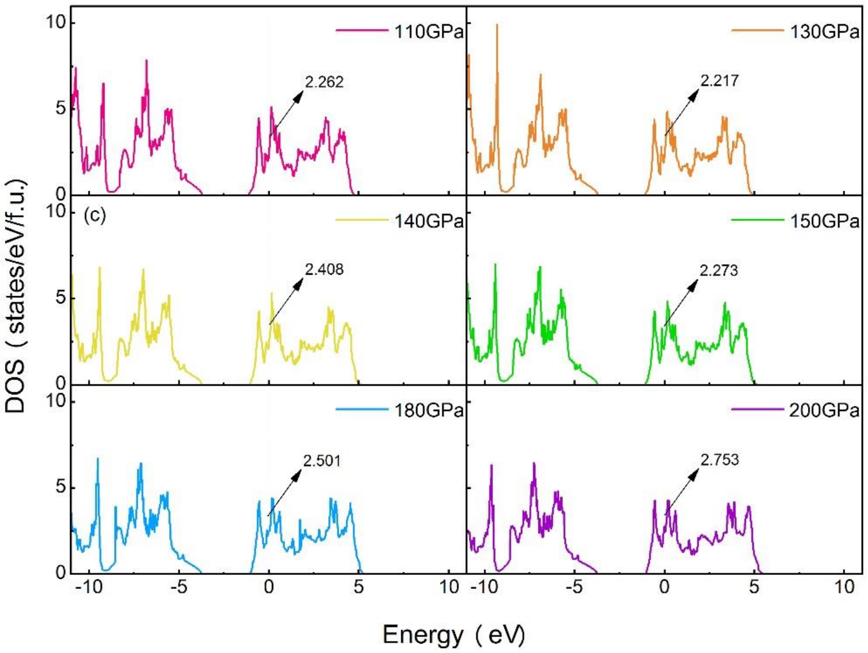

Fig. S25 Calculated total DOS of BaC_{10} at different pressures. Zero energy denotes the Fermi level. The DOS value at Fermi level is present.

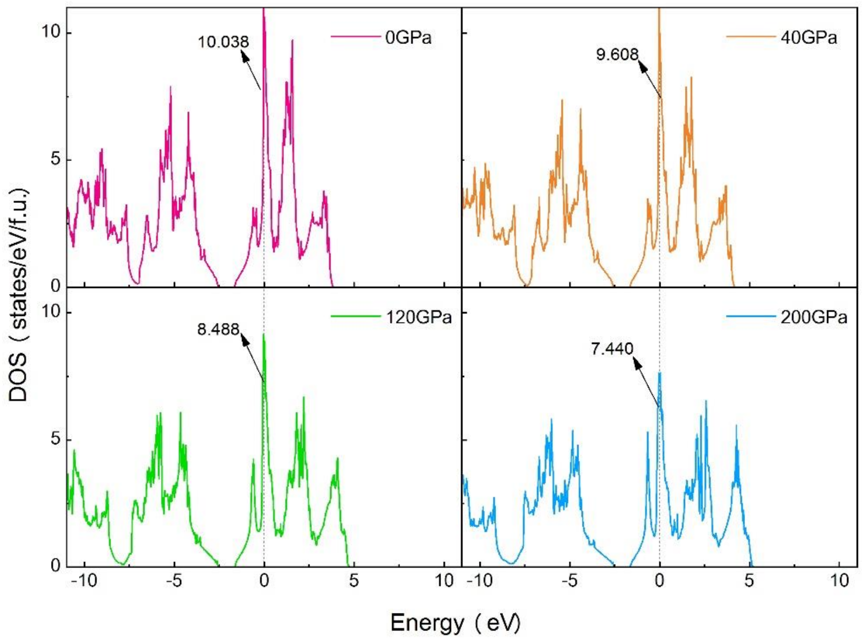

Fig. S26 Calculated total DOS of ScC_{10} at different pressures. Zero energy denotes the Fermi level. The DOS value at Fermi level is present.

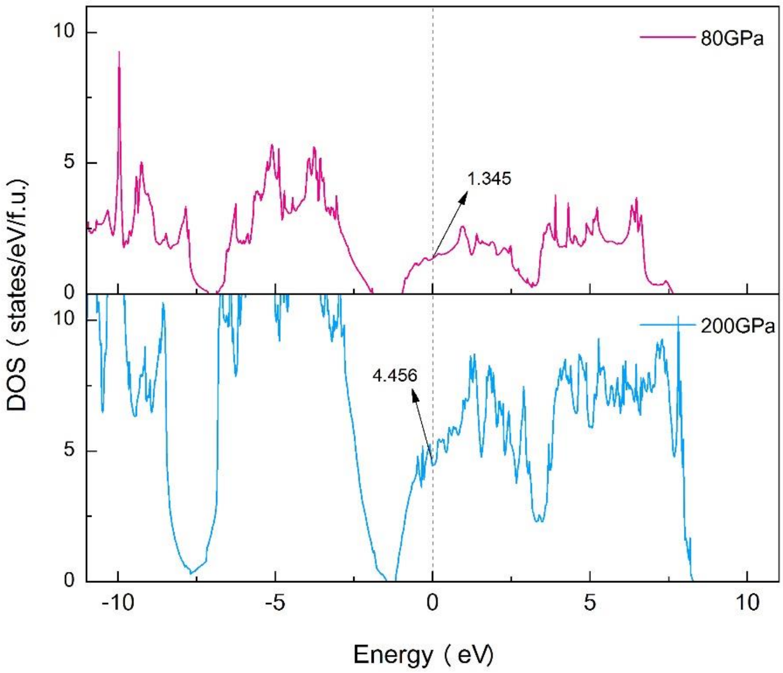

Fig. S27 Calculated total DOS of AlC_{10} at different pressures. Zero energy denotes the Fermi level. The DOS value at Fermi level is present.

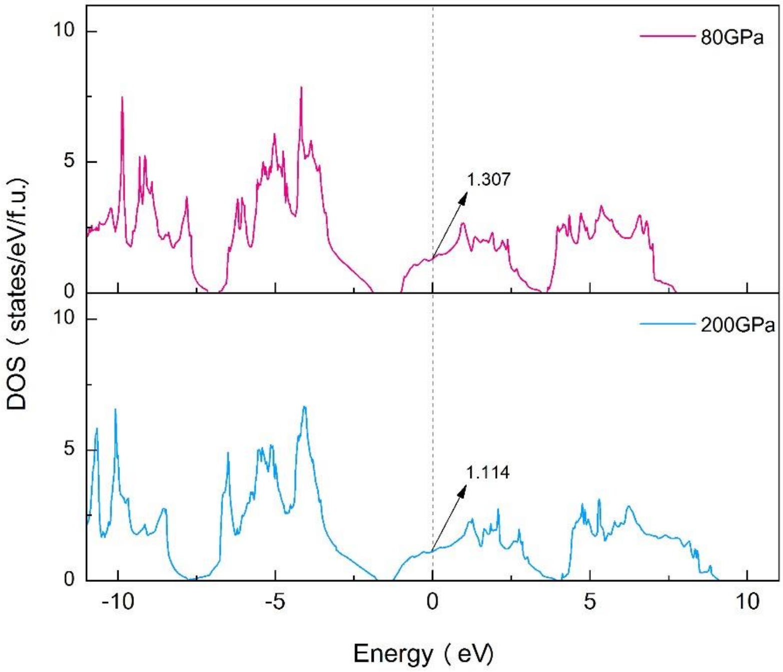

Fig. S28 Calculated total DOS of GaC_{10} at different pressures. Zero energy denotes the Fermi level. The DOS value at Fermi level is present.

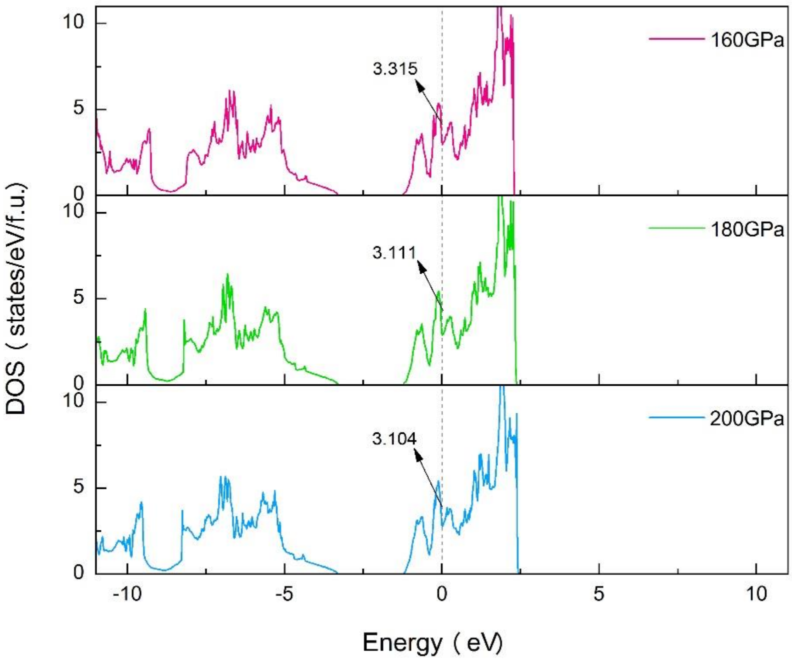

Fig. S29 Calculated total DOS of LaC_{10} at different pressures. Zero energy denotes the Fermi level. The DOS value at Fermi level is present.

3. Calculated phonon spectra of MC_x (x=6 and 10) at different pressures

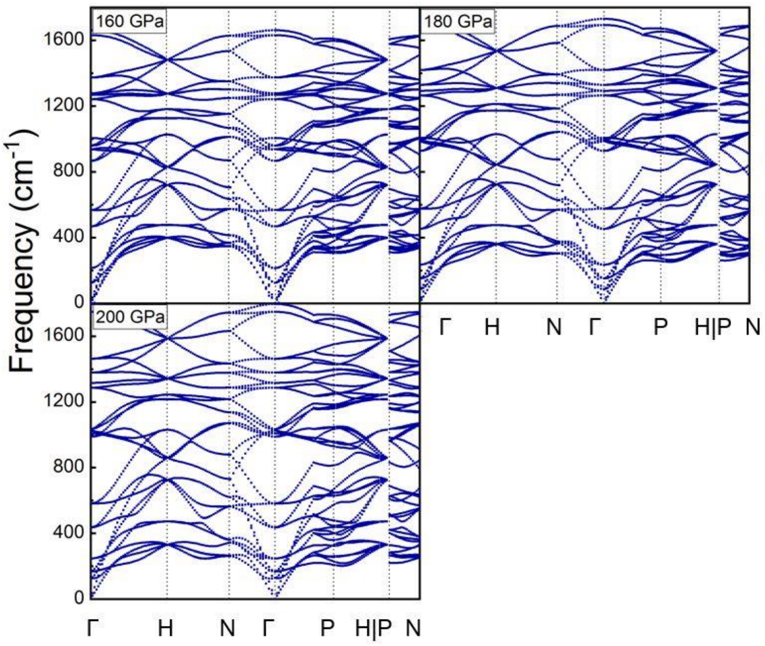

Fig. S30 Calculated phonon spectra along high-symmetrical *k*-point paths of LiC_6 at different pressures.

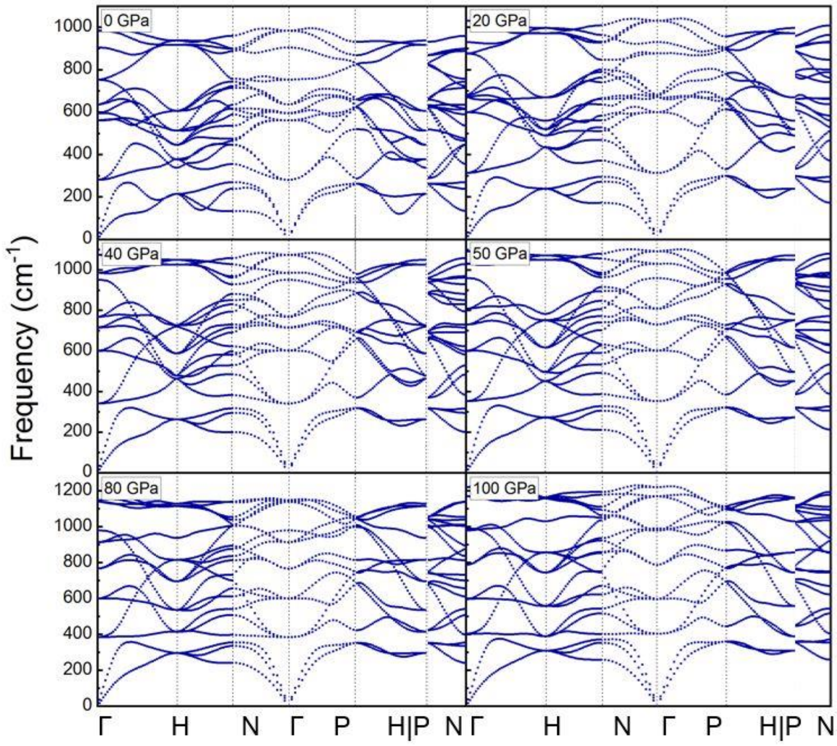

Fig. S31 Calculated phonon spectra along high-symmetrical *k*-point paths of NaC_6 at different pressures.

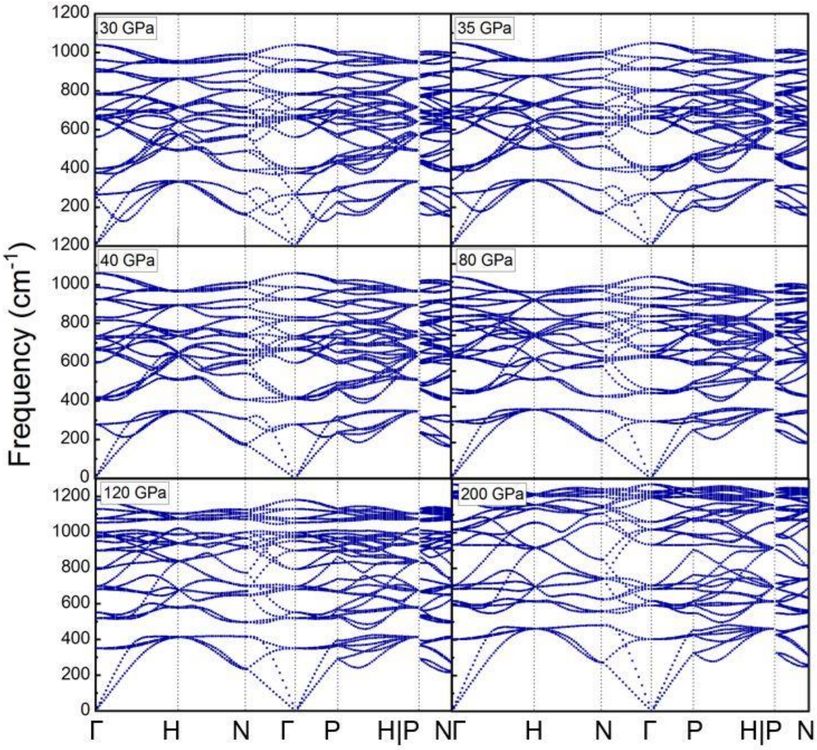

Fig. S32 Calculated phonon spectra along high-symmetrical *k*-point paths of KC_6 at different pressures.

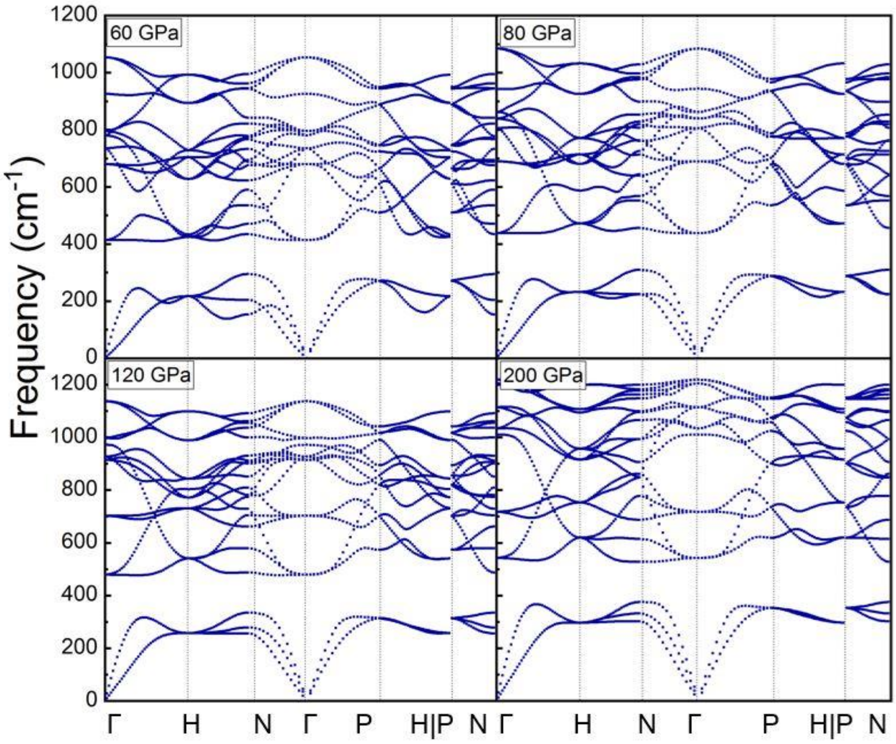

Fig. S33 Calculated phonon spectra along high-symmetrical *k*-point paths of RbC_6 at different pressures.

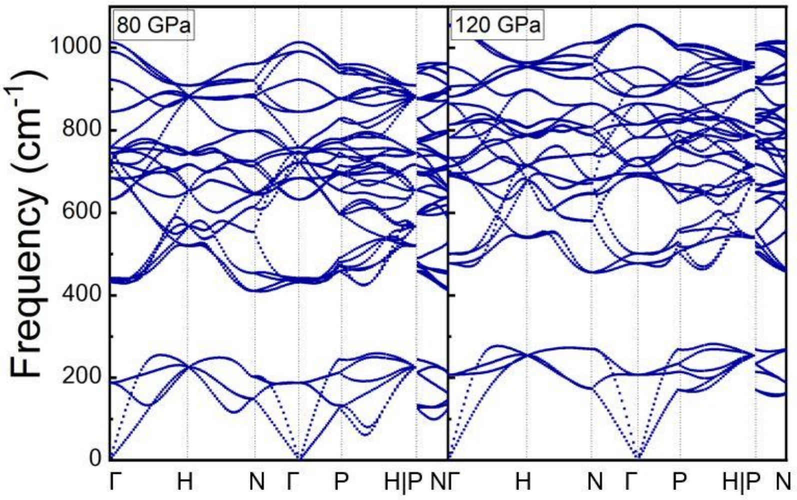

Fig. S34 Calculated phonon spectra along high-symmetrical *k*-point paths of CsC_6 at different pressures.

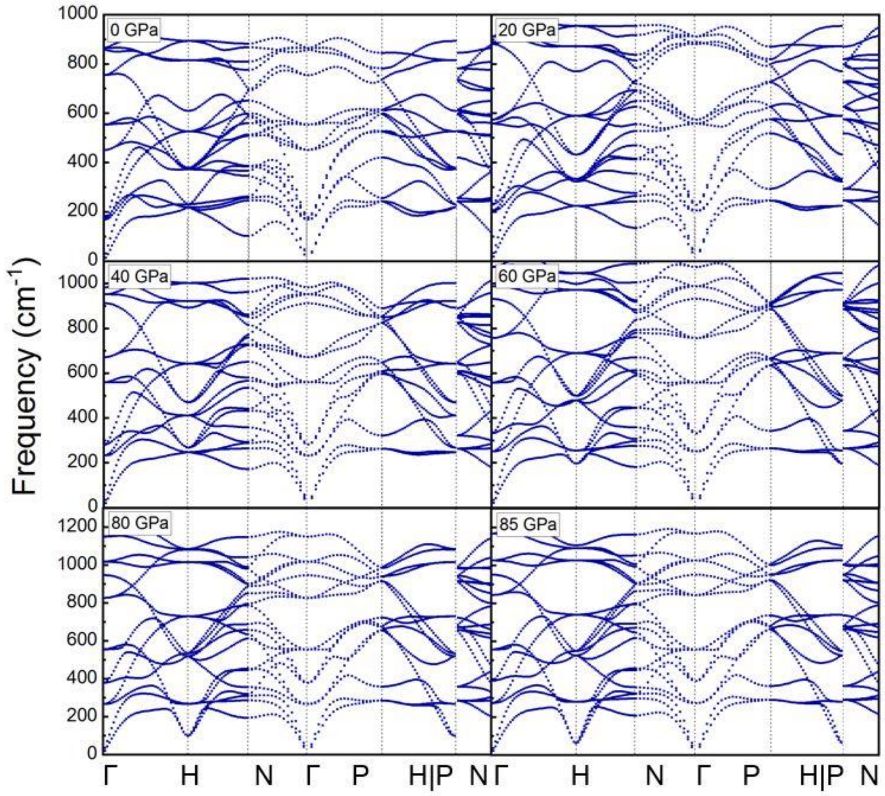

Fig. S35 Calculated phonon spectra along high-symmetrical *k*-point paths of MgC_6 at different pressures.

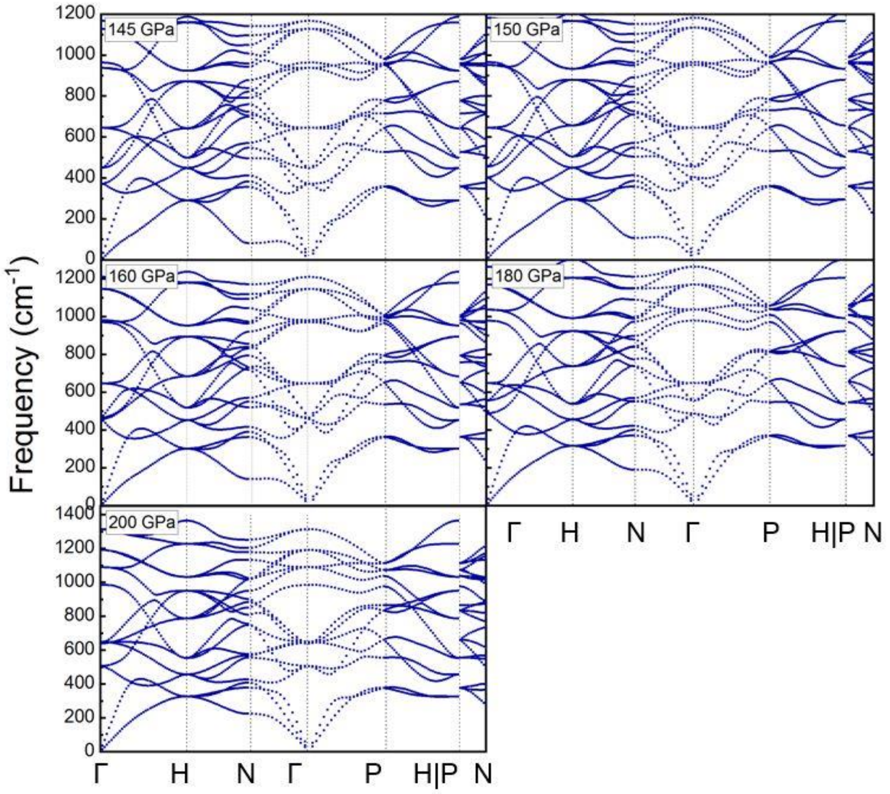

Fig. S36 Calculated phonon spectra along high-symmetrical *k*-point paths of CaC_6 at different pressures.

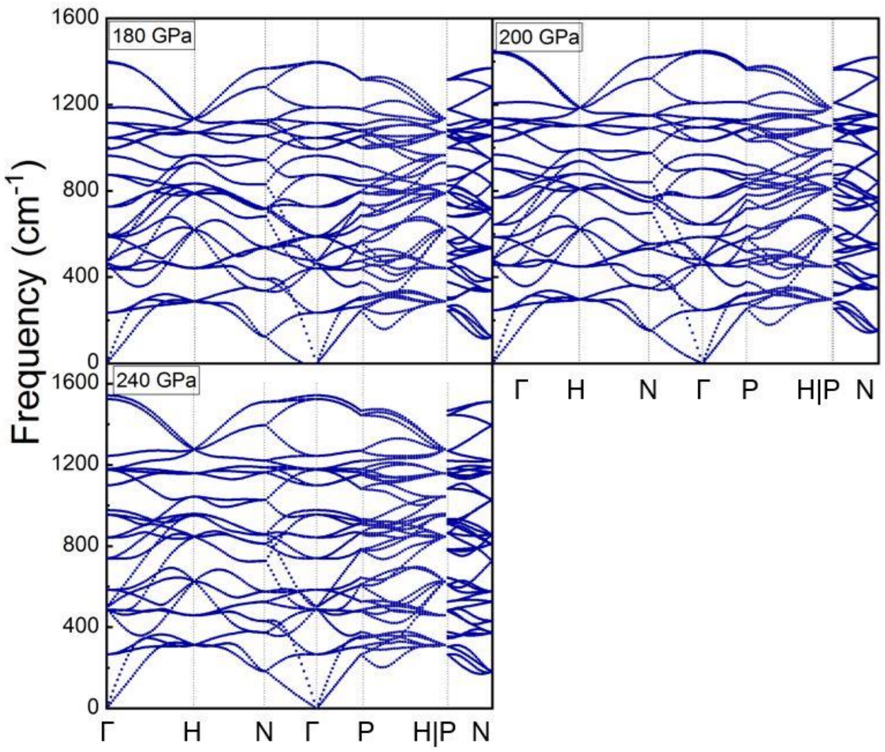

Fig. S37 Calculated phonon spectra along high-symmetrical *k*-point paths of ScC_6 at different pressures.

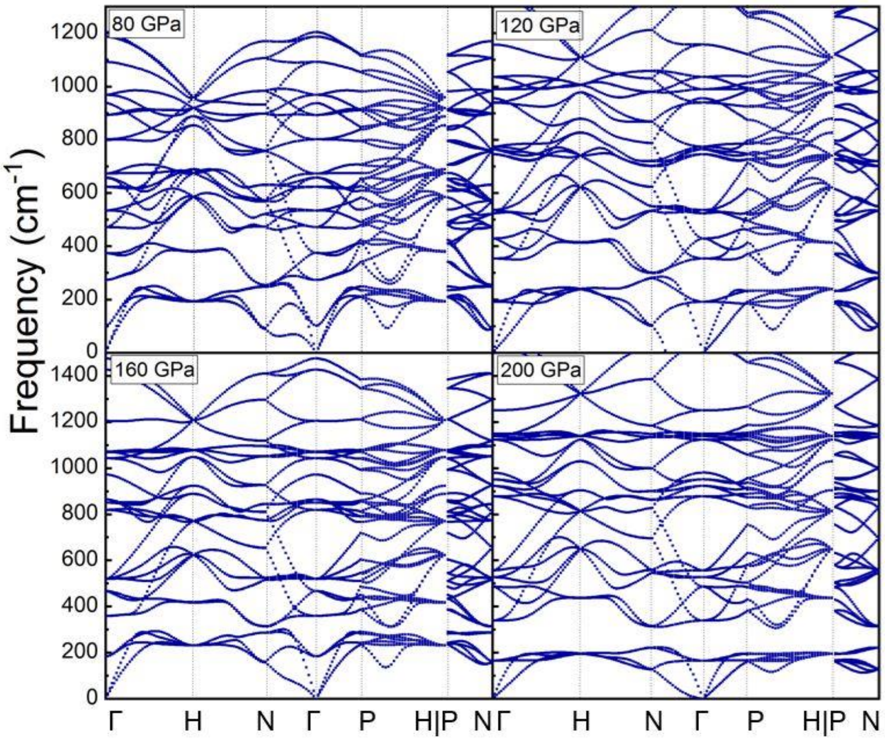

Fig. S38 Calculated phonon spectra along high-symmetrical *k*-point paths of TiC_6 at different pressures.

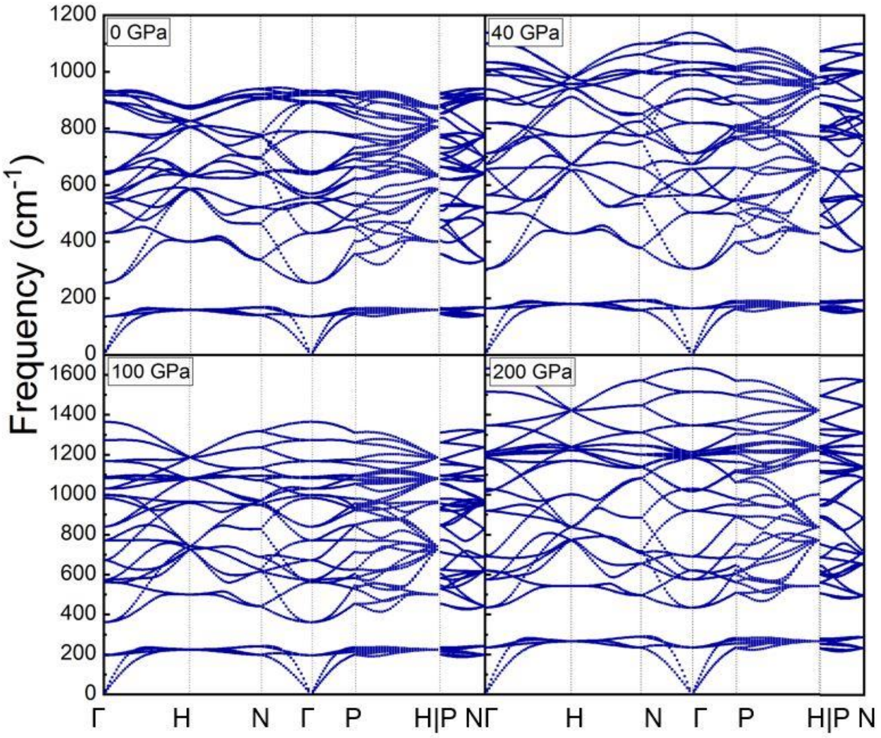

Fig. S39 Calculated phonon spectra along high-symmetrical *k*-point paths of AgC_6 at different pressures.

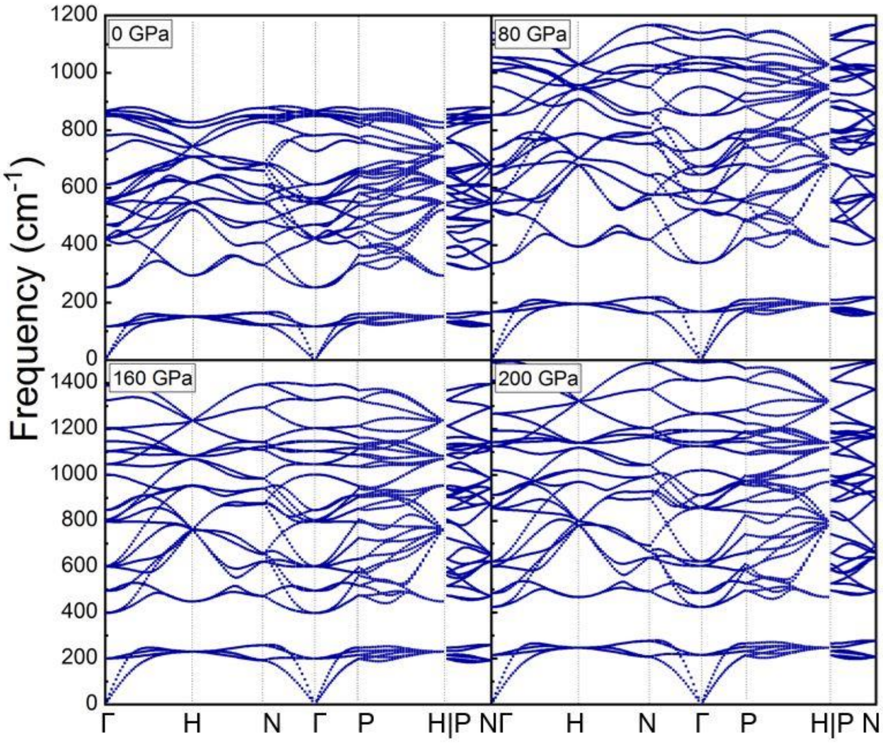

Fig. S40 Calculated phonon spectra along high-symmetrical *k*-point paths of CdC_6 at different pressures.

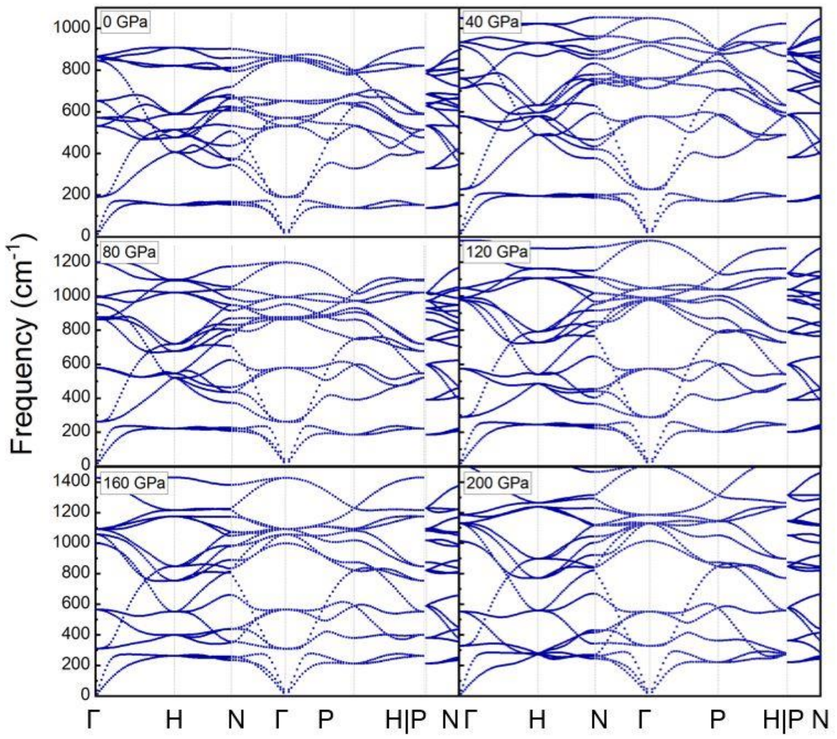

Fig. S41 Calculated phonon spectra along high-symmetrical *k*-point paths of AlC_6 at different pressures.

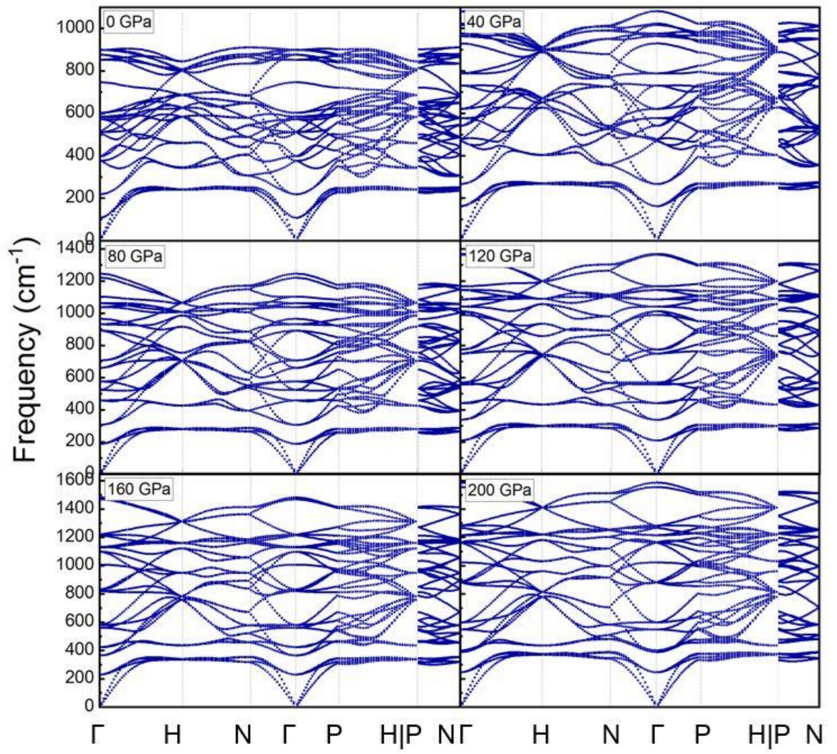

Fig. S42 Calculated phonon spectra along high-symmetrical *k*-point paths of GaC_6 at different pressures.

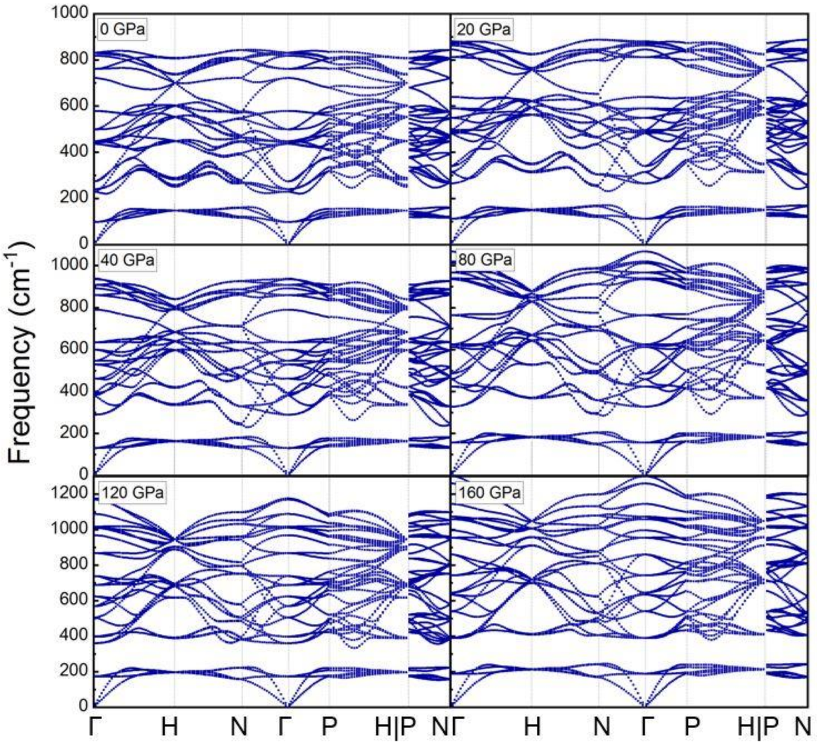

Fig. S43 Calculated phonon spectra along high-symmetrical *k*-point paths of InC_6 at different pressures.

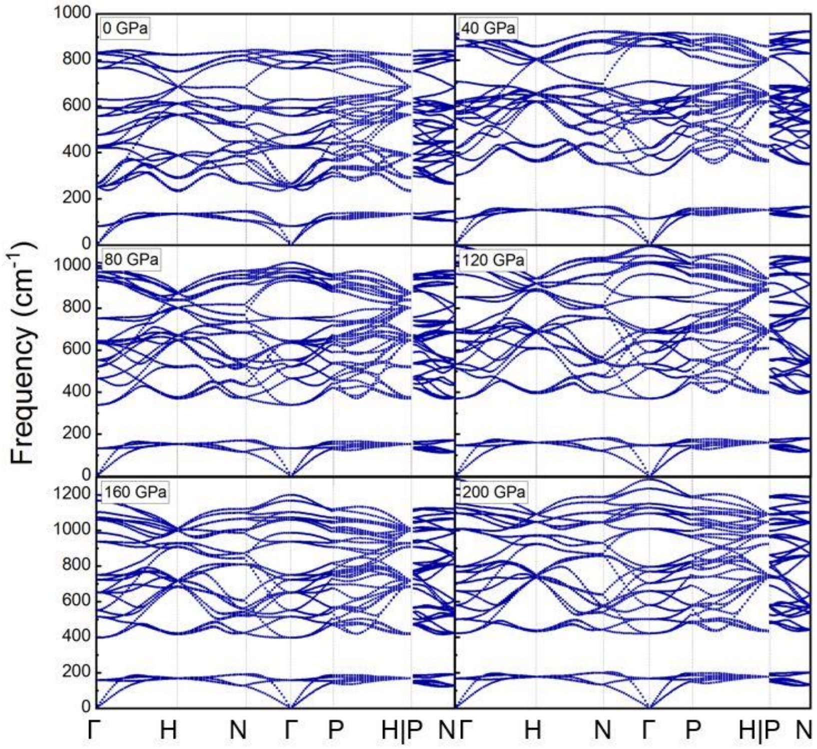

Fig. S44 Calculated phonon spectra along high-symmetrical *k*-point paths of TlC_6 at different pressures.

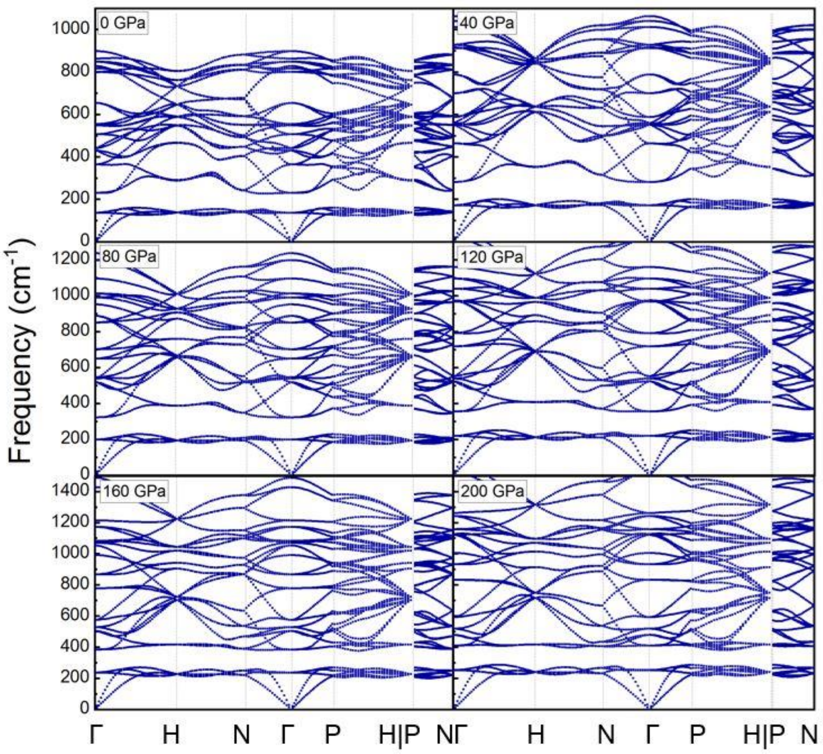

Fig. S45 Calculated phonon spectra along high-symmetrical *k*-point paths of GeC_6 at different pressures.

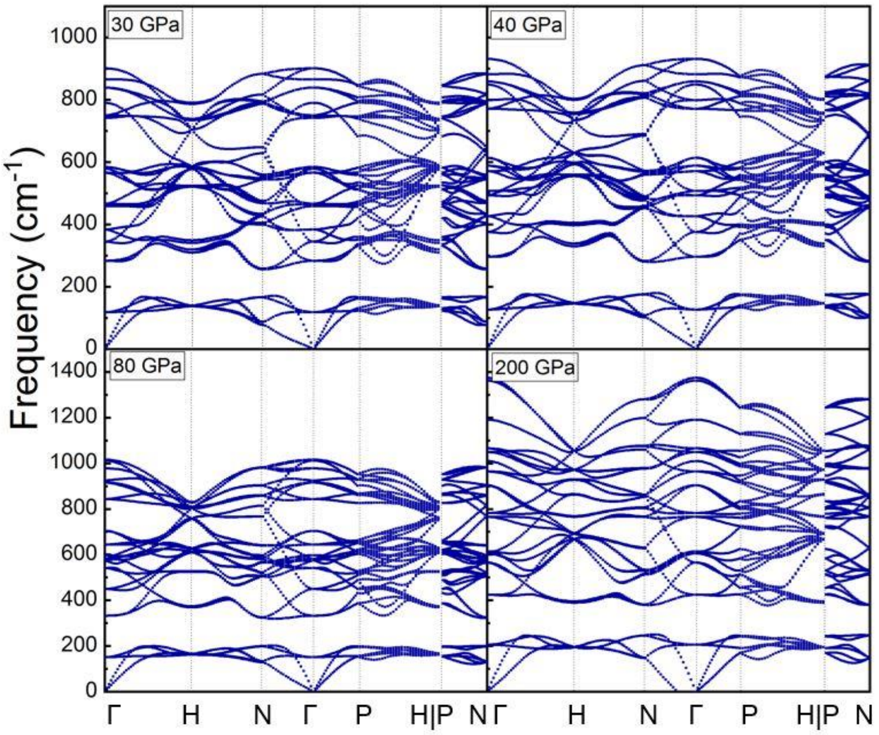

Fig. S46 Calculated phonon spectra along high-symmetrical *k*-point paths of SnC_6 at different pressures.

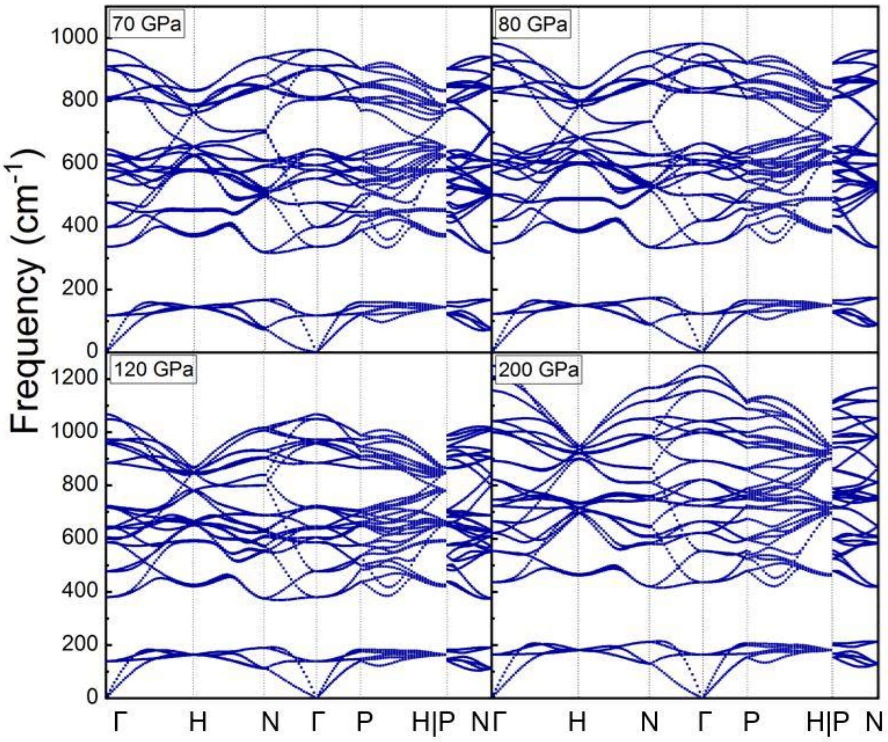

Fig. S47 Calculated phonon spectra along high-symmetrical *k*-point paths of PbC_6 at different pressures.

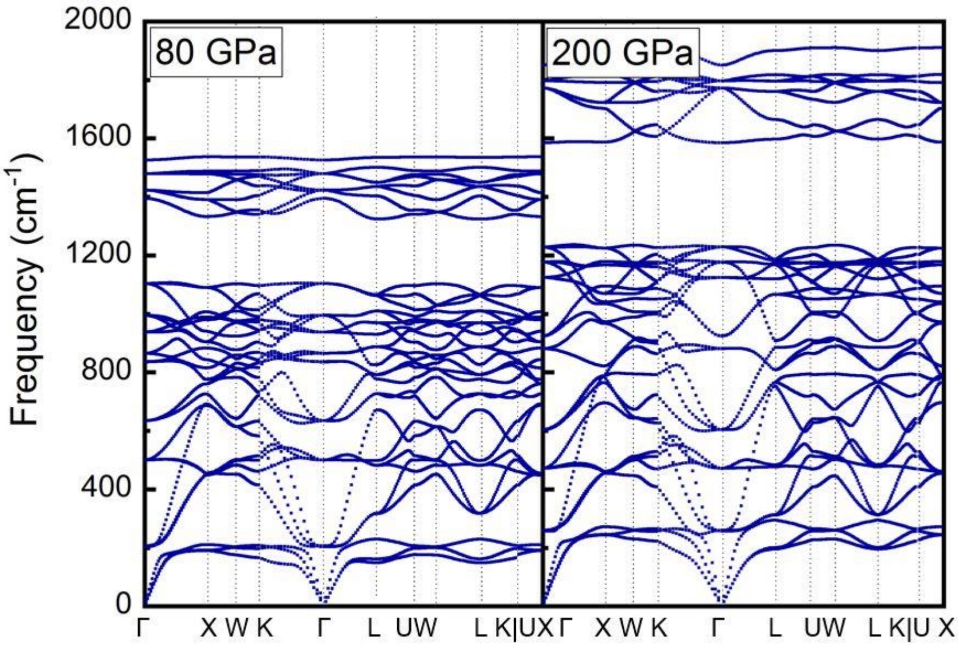

Fig. S48 Calculated phonon spectra along high-symmetrical *k*-point paths of NaC_{10} at different pressures.

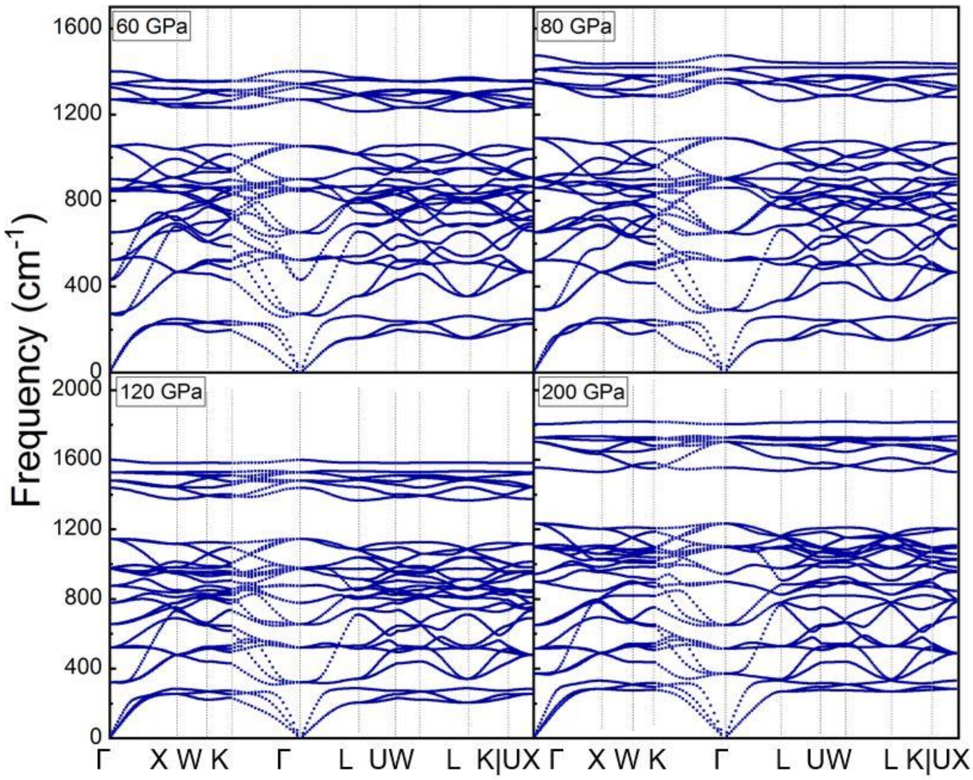

Fig. S49 Calculated phonon spectra along high-symmetrical *k*-point paths of KC_{10} at different pressures.

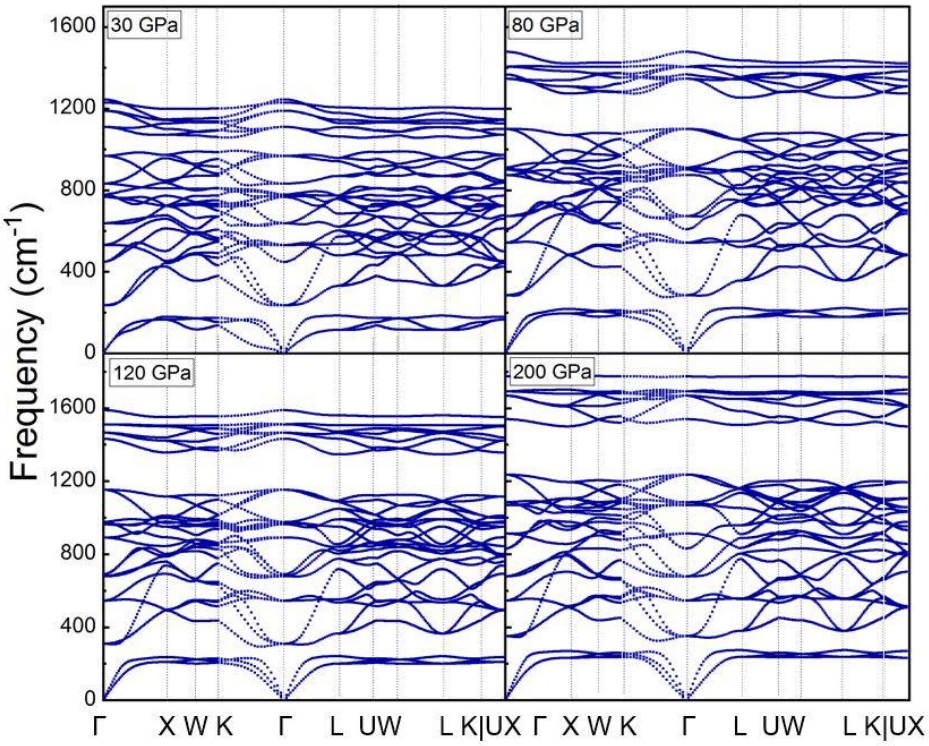

Fig. S50 Calculated phonon spectra along high-symmetrical *k*-point paths of RbC_{10} at different pressures.

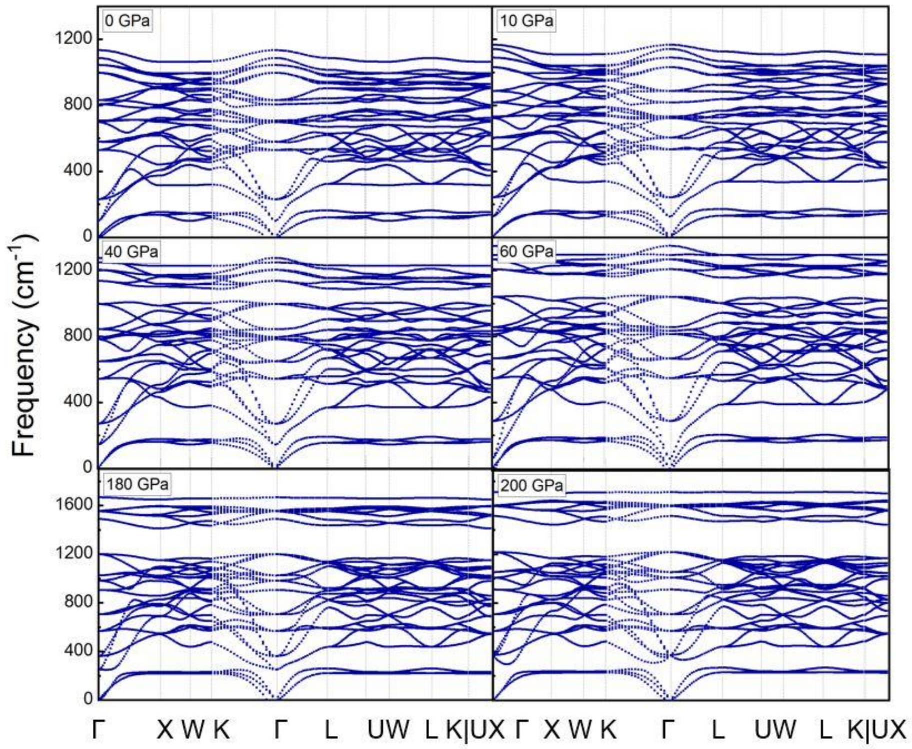

Fig. S51 Calculated phonon spectra along high-symmetrical *k*-point paths of CsC_{10} at different pressures.

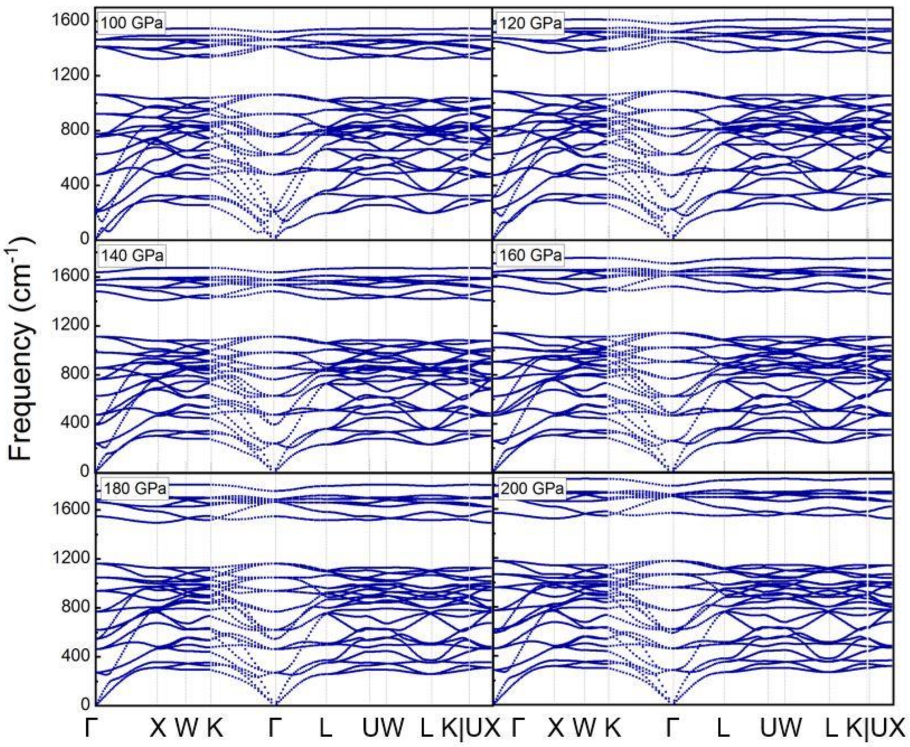

Fig. S52 Calculated phonon spectra along high-symmetrical *k*-point paths of CaC_{10} at different pressures.

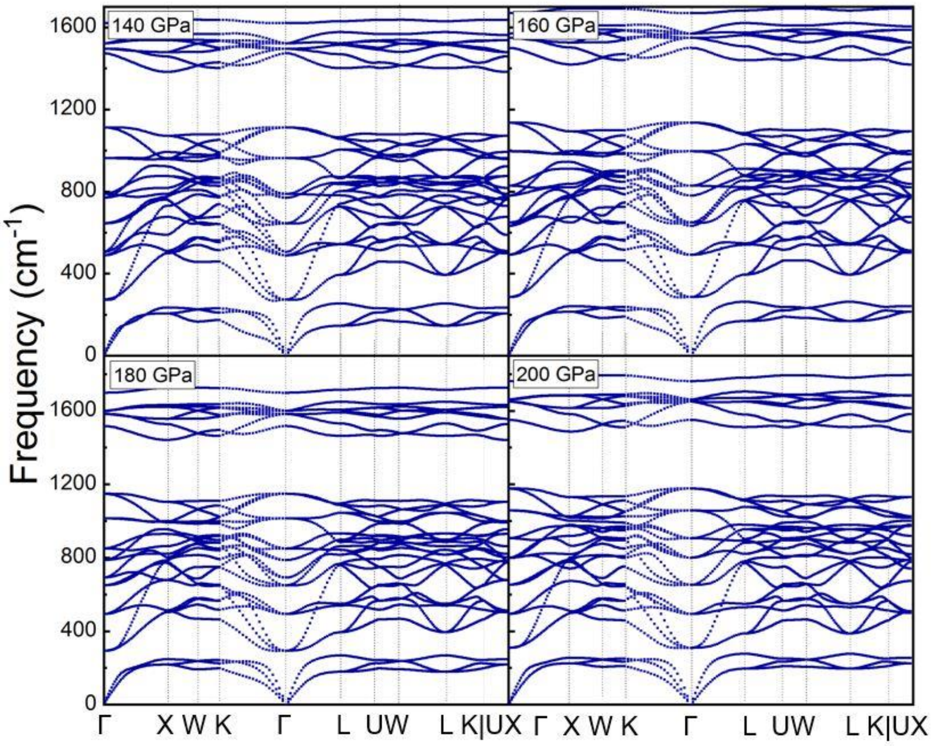

Fig. S53 Calculated phonon spectra along high-symmetrical *k*-point paths of SrC_{10} at different pressures.

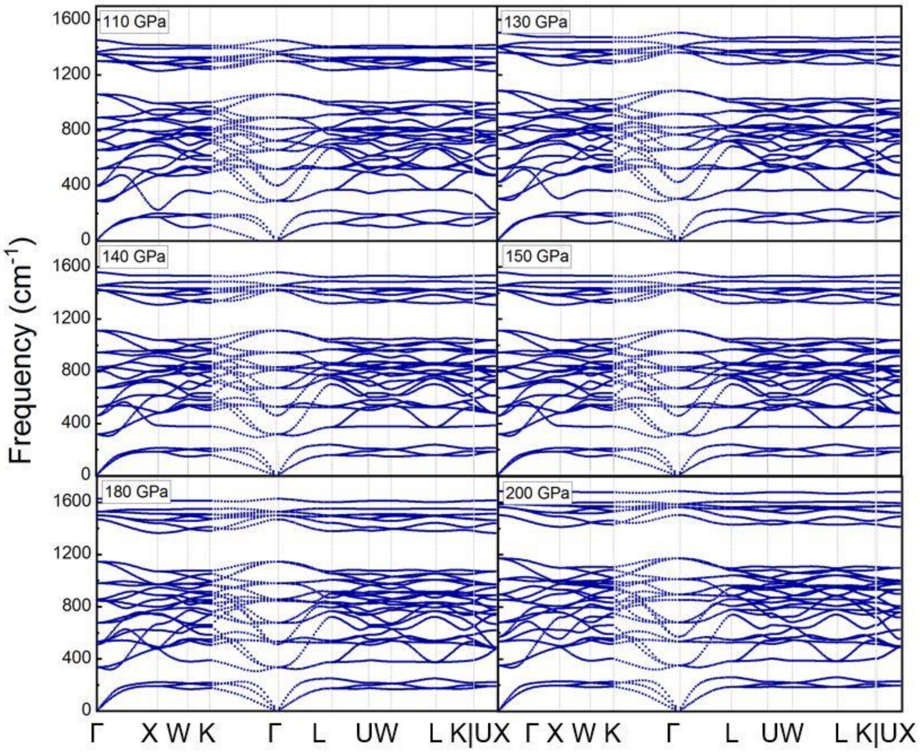

Fig. S54 Calculated phonon spectra along high-symmetrical *k*-point paths of BaC_{10} at different pressures.

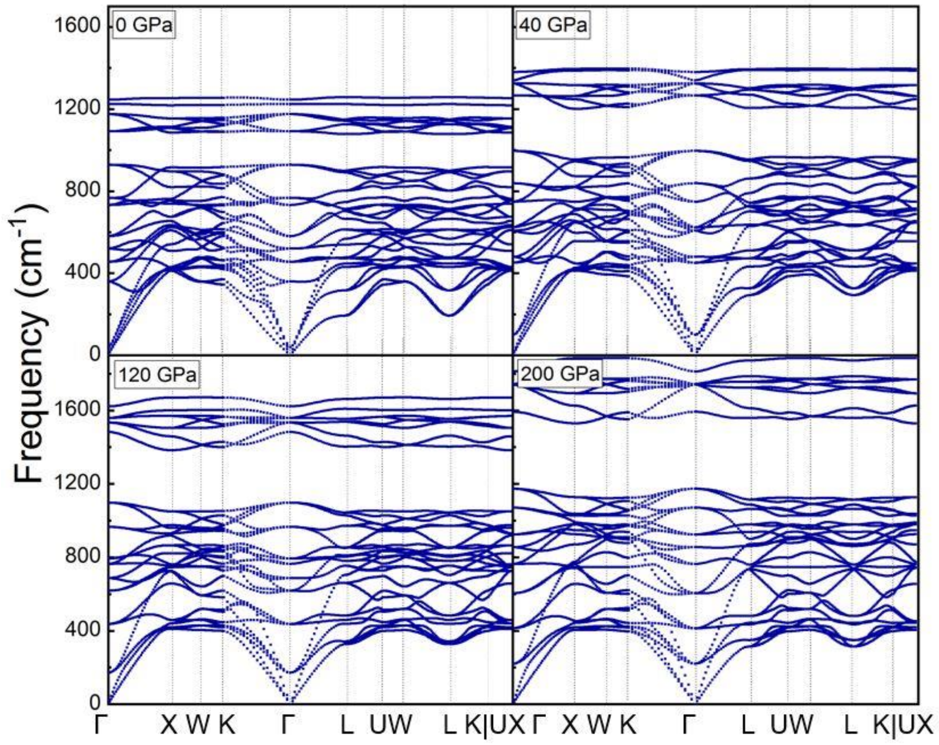

Fig. S55 Calculated phonon spectra along high-symmetrical *k*-point paths of ScC_{10} at different pressures.

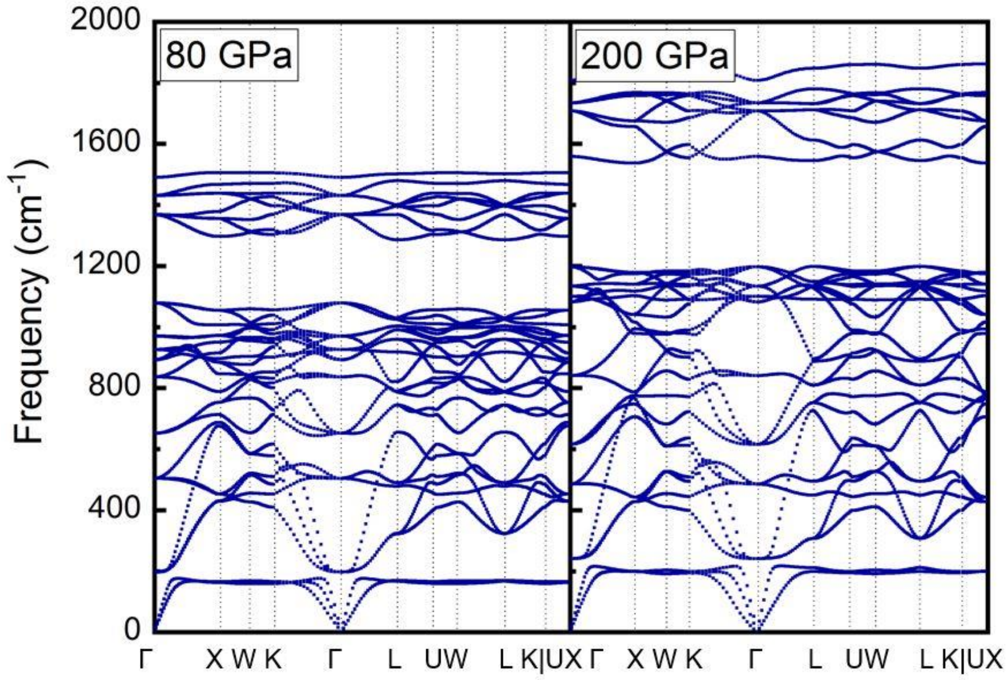

Fig. S56 Calculated phonon spectra along high-symmetrical *k*-point paths of AlC_{10} at different pressures.

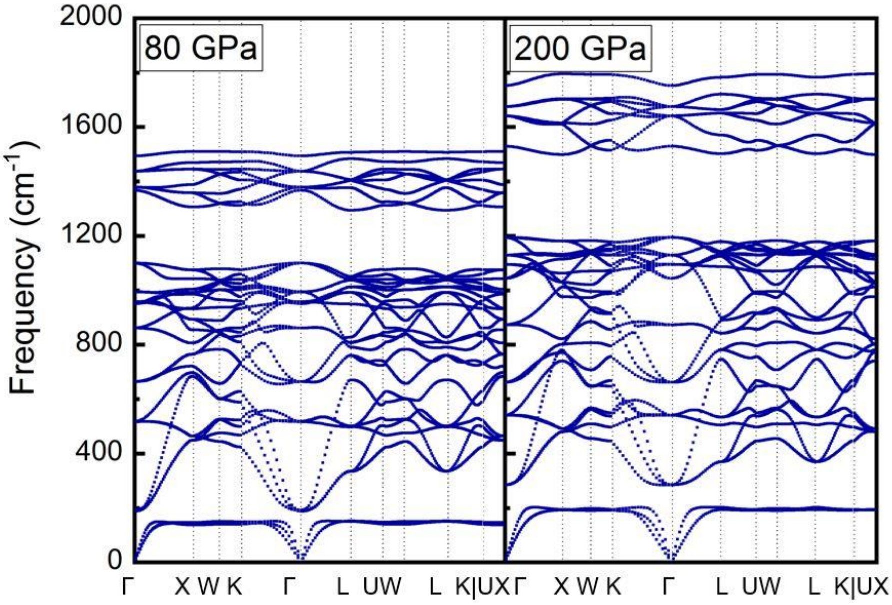

Fig. S57 Calculated phonon spectra along high-symmetrical *k*-point paths of GaC_{10} at different pressures.

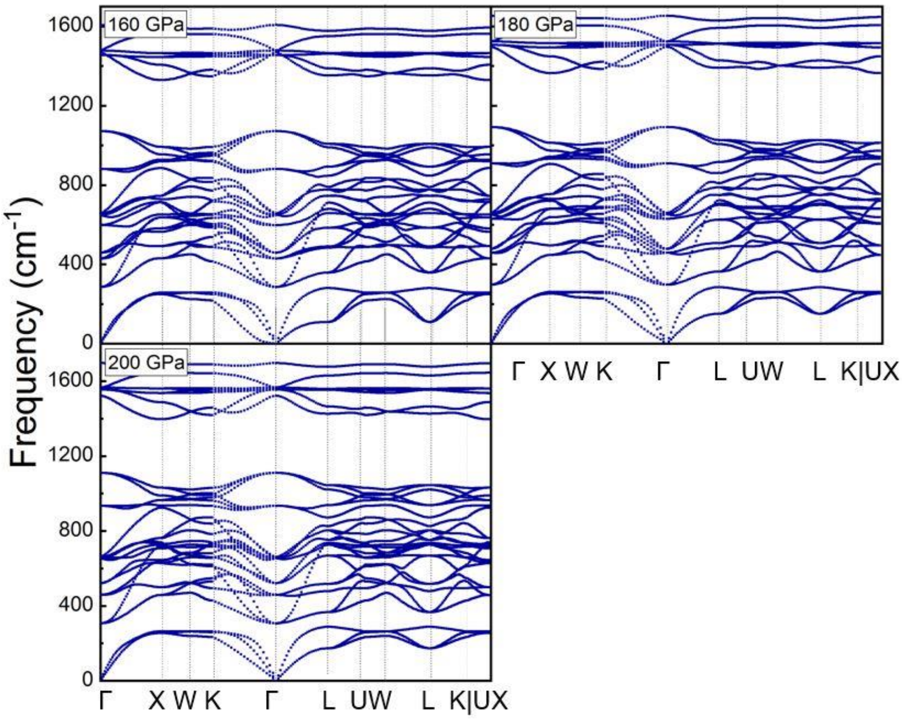

Fig. S58 Calculated phonon spectra along high-symmetrical *k*-point paths of LaC_{10} at different pressures.

4. Electron-phonon coupling constant λ, logarithmic average of phonon frequency ω_{log} (K), and transition temperature T_c (K)

Table S2 Calculated λ, ω_{log}, T_c for each MC_x at different pressures.

System	Pressure (GPa)	λ	ω_{log} (K)	T_c (K)
LiC_6	160	1.93	361.5	65.7
	180	1.80	586.4	96.8
	200	2.05	527.6	101.6
NaC_6	0	2.90	455.3	113.3
	20	1.92	627.4	108.4
	40	1.69	728.6	109.6
	50	1.60	761.3	107.9
	80	1.45	835.2	105.8
	100	1.42	877.5	108.2
KC_6	30	3.07	408.0	109.9
	35	2.28	534.9	110.2
	40	1.93	602.3	104.3
	60	1.35	815.0	93.8
	80	1.19	909.4	88.5
	120	1.01	1034.4	78.6
	160	0.96	1109.6	77.8
	200	0.94	1162.4	78.8
RbC_6	60	2.11	484.6	92.0
	80	1.34	709.8	82.0
	100	1.07	850.6	71.5
	120	0.97	947.5	67.5
	200	0.71	1128.7	42.0
CsC_6	80	3.58	251.5	82.2
	120	1.23	736.9	76.3
MgC_6	0	2.88	449.7	111.6
	20	2.43	541.3	114.2
	40	2.19	616.7	118.0
	60	2.14	637.8	120.0
	80	2.17	642.9	123.3
	85	2.19	637.4	124.2
CaC_6	145	3.16	460.7	131.6
	150	2.75	528.8	129.5
	160	2.33	614.3	126.5
	180	1.86	745.1	122.0

	200	1.57	833.3	116.0
**ScC_6**	180	2.01	459.7	84.3
	200	1.62	639.5	92.1
	240	1.38	750.7	91.1
**TiC_6**	80	1.45	464.1	59.5
	100	1.26	447.4	48.8
	120	1.62	297.1	44.2
	150	1.23	387.1	40.2
	160	1.04	599.4	48.6
	200	0.99	511.7	38.0
**AgC_6**	0	0.43	872.1	5.4
	10	0.38	912.1	2.7
	20	0.30	849.2	0.5
	40	0.28	907.9	0.2
	100	0.29	1157.7	0.4
	150	0.28	1225.6	0.2
	200	0.27	1273.7	0.2
**CdC_6**	0	0.58	706.6	14.9
	10	0.52	771.2	11.4
	50	0.52	812.0	11.6
	80	0.51	873.1	11.8
	100	0.52	905.0	12.8
	120	0.51	947.4	12.9
	160	0.51	980.6	13.4
	200	0.51	1044.7	13.9
**AlC_6**	0	2.15	573.5	110.7
	20	1.89	647.9	109.8
	40	1.67	702.8	104.7
	80	1.41	773.5	95.6
	120	1.43	796.5	100.5
	160	1.45	772.3	100.0
	200	1.78	652.6	105.3
**GaC_6**	0	1.29	700.8	75.3
	40	0.98	840.2	61.0
	80	0.87	902.2	52.3
	120	0.77	972.6	44.0
	160	0.71	1024.7	39.1
	200	0.71	1017.9	38.4
**InC_6**	0	2.32	528.8	104.6
	20	1.92	586.9	98.8
	40	1.79	628.5	99.1
	80	1.57	714.7	97.7

	120	1.42	774.6	94.8
	160	1.24	839.4	87.1
**TlC_6**	0	2.51	490.0	104.0
	20	1.76	610.5	93.8
	40	1.34	737.0	83.5
	80	1.23	793.8	80.4
	120	1.08	851.5	72.4
	160	1.04	901.6	71.9
	200	1.00	944.6	71.4
**GeC_6**	0	1.19	527.3	52.4
	40	0.91	664.3	43.1
	80	0.83	725.6	39.5
	120	0.78	803.3	38.1
	160	0.74	829.9	34.9
	200	0.75	830.6	35.8
**SnC_6**	30	2.01	515.7	93.5
	40	1.76	603.9	94.0
	60	1.52	674.5	89.4
	80	1.39	716.7	85.8
	120	1.27	783.8	83.6
	160	1.32	760.3	86.7
	200	1.35	684.2	79.9
**PbC_6**	70	1.78	556.7	88.5
	75	1.75	599.5	95.1
	80	1.82	648.2	105.2
	90	1.58	684.6	95.6
	100	1.50	721.0	94.1
	120	1.41	772.6	91.6
	160	1.29	849.5	92.9
	200	1.31	898.3	99.4
**NaC_{10}**	80	0.46	747.9	6.3
	200	0.51	842.3	11.1
**KC_{10}**	60	1.10	535.9	47.7
	80	1.41	503.1	63.3
	120	0.93	759.6	50.2
	160	0.79	907.6	44.1
	200	0.68	975.9	33.3
**RbC_{10}**	30	2.11	333.1	66.7
	40	1.49	492.7	66.2
	60	1.12	707.1	64.5
	80	0.94	903.4	61.6
	100	0.81	908.0	46.3

	120	0.9	829.4	52.5
	160	0.73	926.8	37.3
	200	0.67	1073.1	35.3
**CsC_{10}**	0	2.30	346.2	74.3
	5	1.91	443.1	78.1
	10	1.86	511.7	86.6
	20	1.54	588.3	80.0
	30	1.44	625.3	80.6
	40	1.42	639.4	79.0
	50	1.29	686.9	76.6
	60	1.36	637.5	75.1
	180	1.10	825.6	72.9
	200	0.95	953.8	66.2
**CaC_{10}**	100	1.75	412.5	65.7
	120	1.32	589.8	67.5
	140	1.11	679.9	60.6
	160	0.96	744.2	52.4
	180	0.90	805.1	51.4
	200	0.87	847.7	50.0
**SrC_{10}**	140	1.42	539.8	68.6
	160	1.08	679.5	58.8
	180	1.01	733.6	56.4
	200	0.89	852.9	52.6
**BaC_{10}**	110	2.48	328.1	74.8
	120	1.95	452.0	80.6
	130	1.75	486.0	79.3
	140	1.53	594.7	81.7
	150	1.53	597.9	82.4
	160	1.12	892.2	81.3
	180	1.24	716.8	74.8
	200	1.08	811.5	70.1
**ScC_{10}**	0	1.44	455.0	58.1
	40	0.96	652.9	46.1
	80	0.76	777.9	34.9
	120	0.71	861.3	32.1
	160	0.67	900.4	29.8
	200	0.60	910.7	21.5
**AlC_{10}**	80	0.48	565.9	6.1
	200	0.45	561.3	4.3
**GaC_{10}**	80	0.42	532.4	2.9
	200	0.30	905.7	0.5
**LaC_{10}**	160	1.82	426.5	71.0

	180	1.43	628.6	78.8
	200	1.21	737.0	74.5

5. Charge transfer

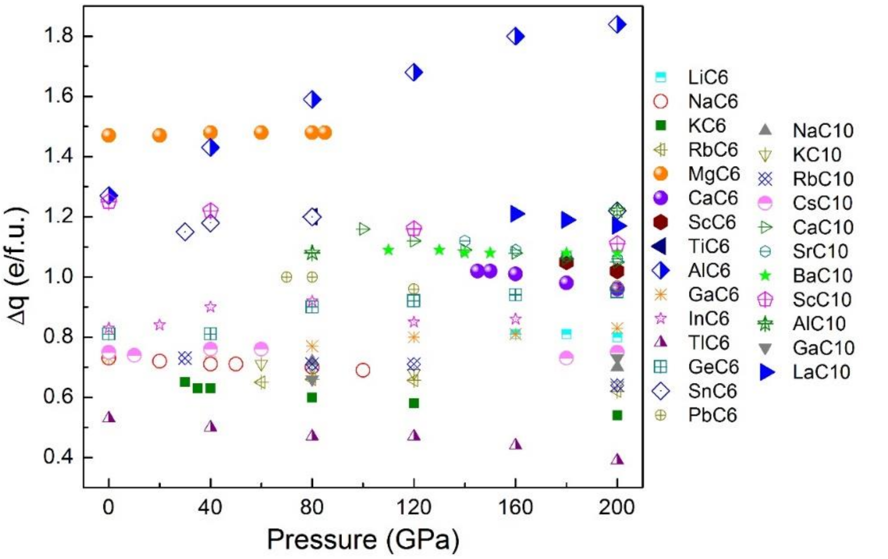

Fig. S59 Calculated transferred charge from metal to C_{24} cage or C_{32} cage.

6. The bonding length of the nearest neighbor C-C

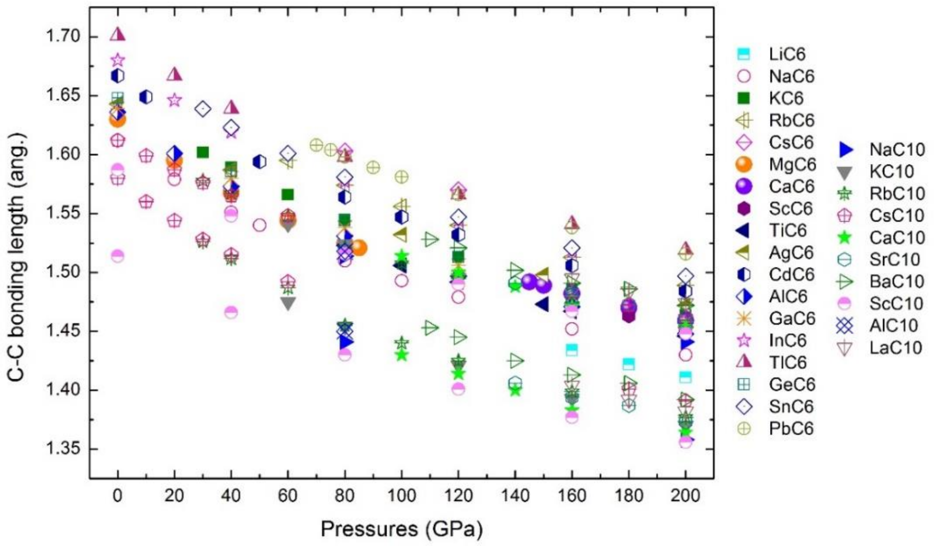

Fig. S60 Calculated the bonding length of the nearest neighbor C-C. In C_{24} cage all the lengths of the nearest neighbor C-C bonds are the same, while in C_{32} cage, there are two kinds of the lengths of the nearest neighbor C-C bonds.

7. 2D ELF comparison between hydrides and carbides

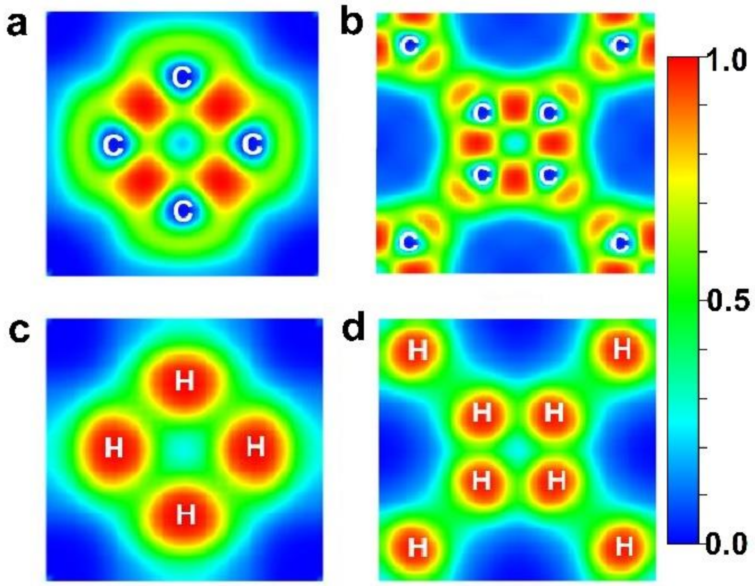

Fig. S61 Calculated 2D electron localization functions (ELF) on (001) plane. **a**, MgC_6 at 0 GPa. **b**, CsC_{10} at 0 GPa. **c**, CaH_6 at 250 GPa. **d**, LaH_{10} at 200 GPa. Stronger hybridization between carbon atoms than between hydrogen atoms.

8. Influence of dopant concentration on T_c for C_{24}-cage-network structures

Table S3. Influence of dopant content on T_c for C_{24}-cage-network structures at ambient pressure.

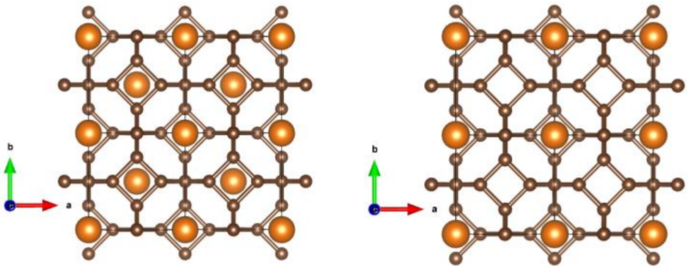

MC_6	T_c (K)	$M_{0.5}C_6$	T_c (K)
NaC_6	113.3	$Na_{0.5}C_6$	46.7
MgC_6	111.6	$Mg_{0.5}C_6$	61.3
AlC_6	110.7	$Al_{0.5}C_6$	49.7
GaC_6	75.3	$Ga_{0.5}C_6$	11.8
InC_6	104.6	$In_{0.5}C_6$	42.6
TlC_6	104.0	$Tl_{0.5}C_6$	53.3
GeC_6	52.4	$Ge_{0.5}C_6$	28.0

9. Defect effect of NaC_6 and CsC_{10} at 0 GPa

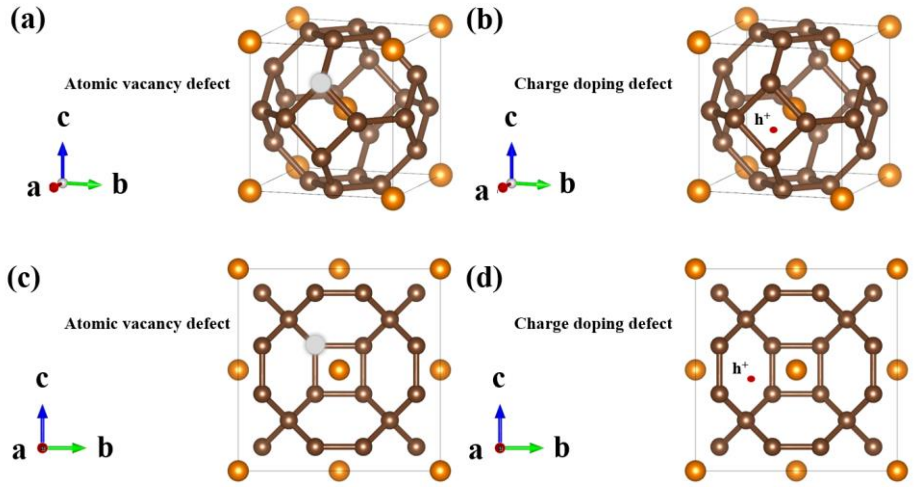

Fig. S62. Schematic diagram of the presence of vacancy defect of C atom and charge doping defects in the system. (a) and (b) are corresponding to NaC_6 with defects, respectively. (c) and (d) are corresponding to CsC_{10} with defects, respectively. Grey ball represents carbon atom vacancy, and the doped charge in system is 0.2 hole per unit cell.

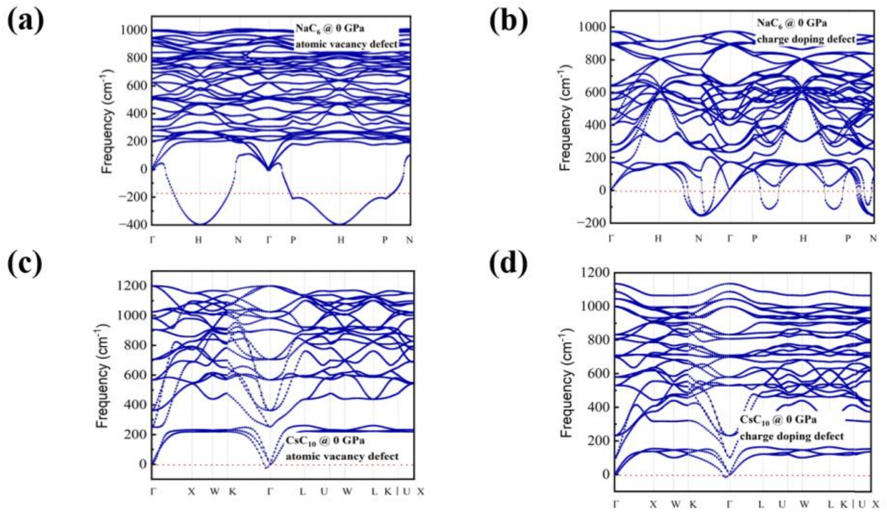

Fig. S63 Calculated phonon spectra along high-symmetrical *k*-point paths at 0 GPa. (a) and (b) are corresponding to NaC_6 with defects, respectively. (c) and (d) are corresponding to CsC_{10} with defects, respectively.

10. Mechanical stability: elastic constants

For MC_6 and MC_{10}, there are 3 independent elastic constants:

C11	C12	C12	0	0	0
C12	C11	C12	0	0	0
C12	C12	C11	0	0	0
0	0	0	C44	0	0
0	0	0	0	C44	0
0	0	0	0	0	C44

Table S4. Calculated elastic constants of MC_6 and MC_{10}, especially for systems that can superconduct at ambient pressure.

System (0 GPa)	Space group	C_{11}	C_{12}	C_{44}	Elastic stability
NaC_6	Im-3m	677.524	57.247	44.666	√
MgC_6	Im-3m	637.056	92.596	7.199	√
AgC_6	Im-3m	733.084	94.453	191.382	√
CdC_6	Im-3m	694.8631	74.0822	113.2485	√
AlC_6	Im-3m	633.0418	94.0788	24.5661	√
GaC_6	Im-3m	673.009	73.846	56.147	√
InC_6	Im-3m	594.402	94.626	28.149	√
TlC_6	Im-3m	545.450	125.580	15.656	√
GeC_6	Im-3m	601.976	119.194	46.613	√
CsC_{10}	Fm-3m	293.947	201.925	232.143	√
ScC_{10}	Fm-3m	316.676	246.562	260.658	√

Based on the elastic stability criteria:

Criteria (i) $C_{11} - C_{12} > 0$

Criteria (ii) $C_{11} + 2C_{12} > 0$

Criteria (iii) $C_{44} > 0$,

we found that these structures are mechanically stable.

11. Thermodynamic stability: enthalpy of formation

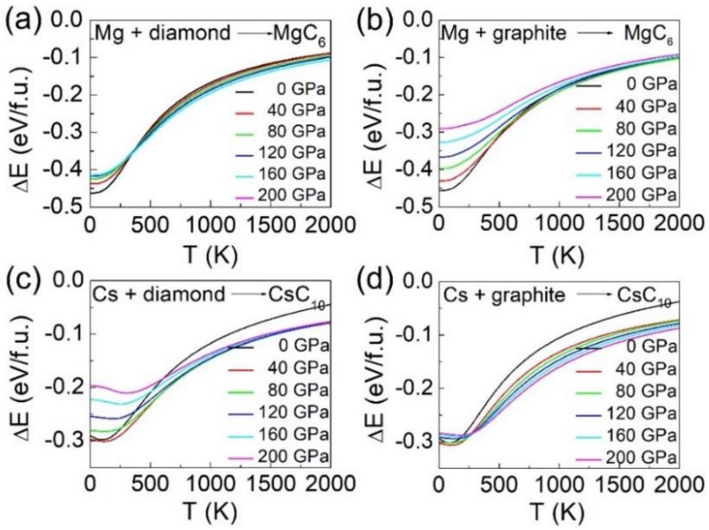

Fig. S64 Enthalpy of formation as a function of pressure and temperature. The enthalpy of formation of MgC_6 [(a), (b)] and CsC_{10} [(c), (d)] were calculated by the QHA method when existing the possibly synthesizing or decomposing routes of metal + diamond and metal + graphite..

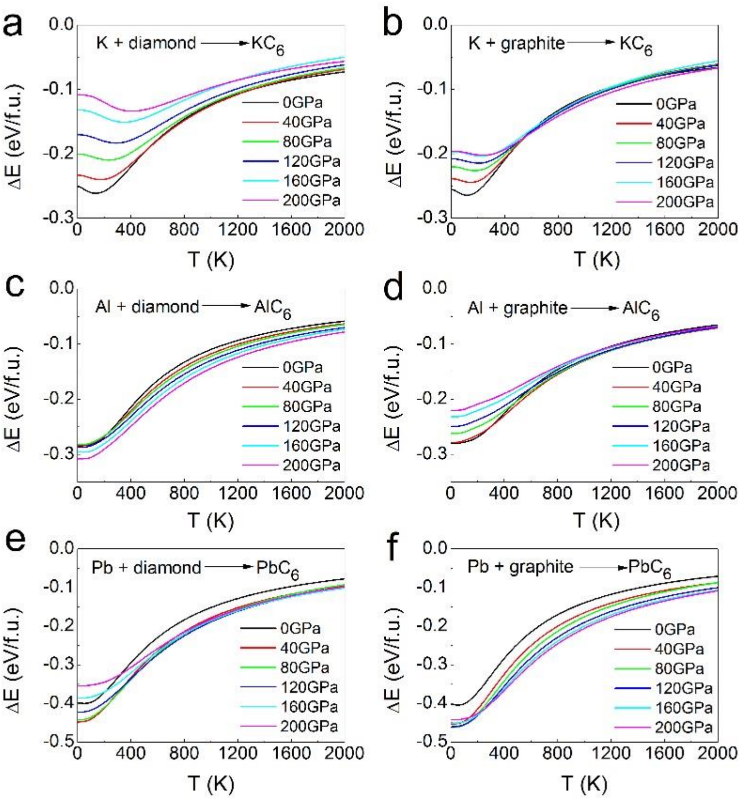

Fig. S65 The enthalpy of formation of KC_6 (**a**, **b**), AlC_6 (**c**, **d**), and PbC_6 (**e**, **f**) were calculated within the quasi-harmonic approximations (QHA) when existing the possible decompositions of metal + diamond and metal + graphite.

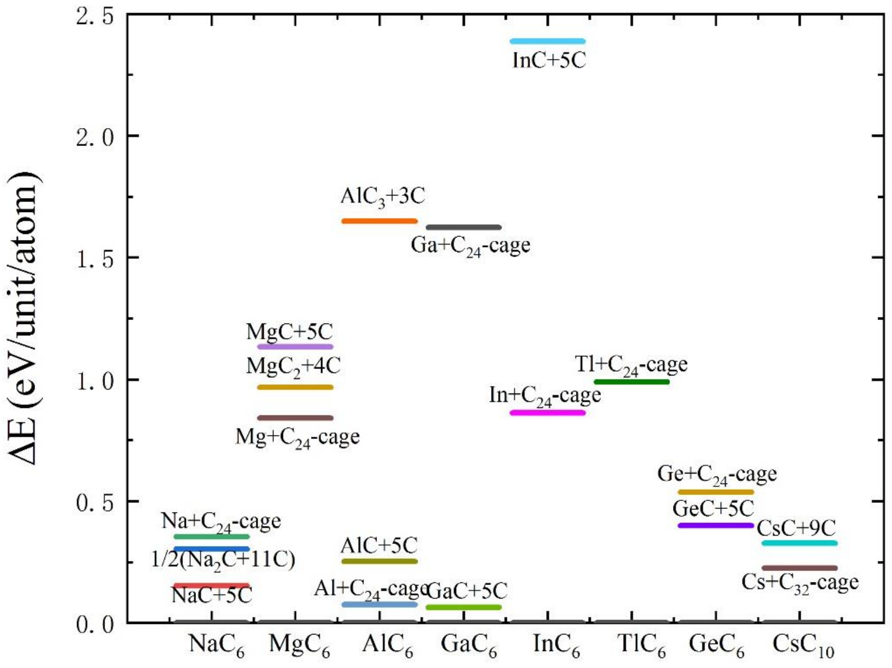

Fig. S66 The relative enthalpy of formation is calculated when MC_6 and MC_{10} are formed through combining different compounds. The enthalpy of MC_6 and MC_{10} is set as the reference point, respectively. The results not only show that MC_6 and MC_{10} have good thermodynamic stability, but also provides some possible synthesis routes.

12. The size dependence of the calculation

We have considered the size dependence of the calculation, taking the superconducting systems at 0 GPa as examples. Two cell sizes have been considered, primitive cell and standard conventional cell, as shown in the following Fig. S67. The following Table S5 presents the calculated superconducting parameters for two kinds of cell sizes. The results show that the dependence of superconducting parameters on cell size is insensitive.

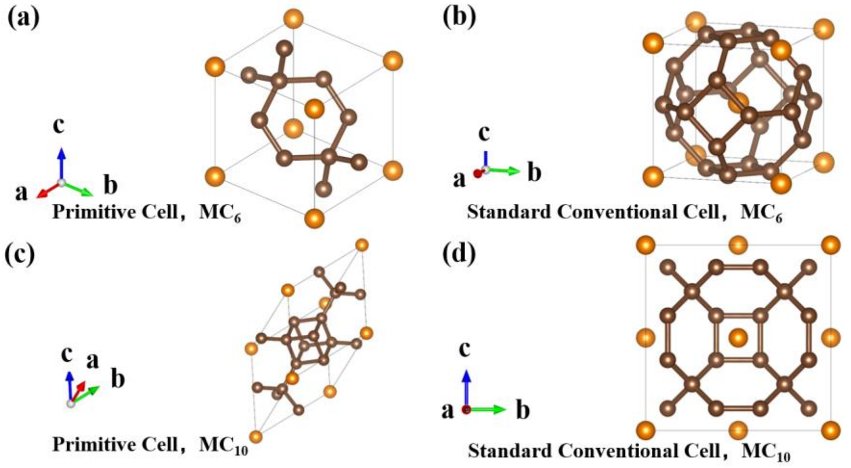

Fig. S67 (a) and (b) are corresponding to the primitive cell and standard convention cell of $M\mathrm{C}_6$, respectively. (c) and (d) are corresponding to the primitive cell and standard convention cell of $M\mathrm{C}_{10}$, respectively.

Table S5 Calculated λ, $\omega_{\log}$, T_c for each $M\mathrm{C}_6$ and $M\mathrm{C}_{10}$ in forms of primitive cell and standard conventional cell at 0 GPa.

System (0 GPa)	Cell size	$\omega_{\log}$ (K)	λ	T_c (k)
NaC_6	Primitive Cell	454.9	2.84	114.3
	Standard Conventional Cell	455.3	2.90	113.3
MgC_6	Primitive Cell	470.6	2.78	113.9
	Standard Conventional Cell	449.7	2.88	111.6
AgC_6	Primitive Cell	832.2	0.38	5.5
	Standard Conventional Cell	872.1	0.43	5.4
CdC_6	Primitive Cell	754.6	0.49	14.3
	Standard Conventional Cell	706.6	0.58	14.9
AlC_6	Primitive Cell	564.5	2.24	112.8

	Standard Conventional Cell	573.5	2.15	110.7
GaC_6	Primitive Cell	650.6	1.37	76.7
	Standard Conventional Cell	700.8	1.29	75.3
InC_6	Primitive Cell	541.5	2.30	106.6
	Standard Conventional Cell	528.8	2.32	104.6
TlC_6	Primitive Cell	466.3	2.48	108.9
	Standard Conventional Cell	490.0	2.51	104.0
GeC_6	Primitive Cell	542.2	1.25	54.3
	Standard Conventional Cell	527.3	1.19	52.4
CsC_{10}	Primitive Cell	346.2	2.30	74.3
	Standard Conventional Cell	326.4	2.27	73.2
ScC_{10}	Primitive Cell	455.0	1.44	58.1
	Standard Conventional Cell	434.9	1.48	57.8

13. POSCAR files of $\boldsymbol{Im\bar{3}m}$ – MC$_6$

NaC_6 at 0 GPa

```
      1.00000000000000
       4.5583697619954391    0.0000000000000000    0.0000000000000000
       0.0000000000000000    4.5583697619954391    0.0000000000000000
       0.0000000000000000    0.0000000000000000    4.5583697619954417
       Na    C
       2     12
Direct
   0.0000000000000000  0.0000000000000000  0.0000000000000000
   0.5000000000000000  0.5000000000000000  0.5000000000000000
   0.2500000000000000  0.0000000000000000  0.5000000000000000
   0.7500000000000000  0.0000000000000000  0.5000000000000000
   0.5000000000000000  0.2500000000000000  0.0000000000000000
   0.5000000000000000  0.7500000000000000  0.0000000000000000
   0.0000000000000000  0.5000000000000000  0.2500000000000000
   0.0000000000000000  0.5000000000000000  0.7500000000000000
   0.0000000000000000  0.2500000000000000  0.5000000000000000
   0.0000000000000000  0.7500000000000000  0.5000000000000000
   0.2500000000000000  0.5000000000000000  0.0000000000000000
   0.7500000000000000  0.5000000000000000  0.0000000000000000
   0.5000000000000000  0.0000000000000000  0.7500000000000000
   0.5000000000000000  0.0000000000000000  0.2500000000000000
```

MgC_6 at 0 GPa

```
     1.00000000000000
       4.6097866790150492    0.0000000000000000    0.0000000000000000
       0.0000000000000000    4.6097866790150483    0.0000000000000000
       0.0000000000000000    0.0000000000000000    4.6097866790150492
     Mg    C
       2     12
Direct
   0.0000000000000000  0.0000000000000000  0.0000000000000000
   0.5000000000000000  0.5000000000000000  0.5000000000000000
   0.2500000000000000  0.0000000000000000  0.5000000000000000
   0.7500000000000000  0.0000000000000000  0.5000000000000000
   0.5000000000000000  0.2500000000000000  0.0000000000000000
   0.5000000000000000  0.7500000000000000  0.0000000000000000
```

```
  0.0000000000000000   0.5000000000000000   0.2500000000000000
  0.0000000000000000   0.5000000000000000   0.7500000000000000
  0.0000000000000000   0.2500000000000000   0.5000000000000000
  0.0000000000000000   0.7500000000000000   0.5000000000000000
  0.2500000000000000   0.5000000000000000   0.0000000000000000
  0.7500000000000000   0.5000000000000000   0.0000000000000000
  0.5000000000000000   0.0000000000000000   0.7500000000000000
  0.5000000000000000   0.0000000000000000   0.2500000000000000
```

AlC_6 at 0 GPa

```
    1.00000000000000
     4.6269276925151530     0.0000000000000000     0.0000000000000000
     0.0000000000000000     4.6269276925151521     0.0000000000000000
     0.0000000000000000     0.0000000000000000     4.6269276925151530
   Al   C
     2    12
Direct
  0.0000000000000000   0.0000000000000000   0.0000000000000000
  0.5000000000000000   0.5000000000000000   0.5000000000000000
  0.2500000000000000   0.0000000000000000   0.5000000000000000
  0.7500000000000000   0.0000000000000000   0.5000000000000000
  0.5000000000000000   0.2500000000000000   0.0000000000000000
  0.5000000000000000   0.7500000000000000   0.0000000000000000
  0.0000000000000000   0.5000000000000000   0.2500000000000000
  0.0000000000000000   0.5000000000000000   0.7500000000000000
  0.0000000000000000   0.2500000000000000   0.5000000000000000
  0.0000000000000000   0.7500000000000000   0.5000000000000000
  0.2500000000000000   0.5000000000000000   0.0000000000000000
  0.7500000000000000   0.5000000000000000   0.0000000000000000
  0.5000000000000000   0.0000000000000000   0.7500000000000000
  0.5000000000000000   0.0000000000000000   0.2500000000000000
```

InC_6 at 0 GPa

```
   1.00000000000000
     4.7519949107220585     0.0000000000000000     0.0000000000000000
     0.0000000000000000     4.7519949107220576     0.0000000000000000
     0.0000000000000000     0.0000000000000000     4.7519949107220585
   In   C
     2    12
Direct
  0.0000000000000000   0.0000000000000000   0.0000000000000000
  0.5000000000000000   0.5000000000000000   0.5000000000000000
  0.2500000000000000   0.0000000000000000   0.5000000000000000
  0.7500000000000000   0.0000000000000000   0.5000000000000000
  0.5000000000000000   0.2500000000000000   0.0000000000000000
  0.5000000000000000   0.7500000000000000   0.0000000000000000
  0.0000000000000000   0.5000000000000000   0.2500000000000000
  0.0000000000000000   0.5000000000000000   0.7500000000000000
  0.0000000000000000   0.2500000000000000   0.5000000000000000
  0.0000000000000000   0.7500000000000000   0.5000000000000000
  0.2500000000000000   0.5000000000000000   0.0000000000000000
  0.7500000000000000   0.5000000000000000   0.0000000000000000
  0.5000000000000000   0.0000000000000000   0.7500000000000000
  0.5000000000000000   0.0000000000000000   0.2500000000000000
```

TlC_6 at 0 GPa

```
    1.00000000000000
     4.8112110347424784     0.0000000000000000     0.0000000000000000
     0.0000000000000000     4.8112110347424775     0.0000000000000000
     0.0000000000000000     0.0000000000000000     4.8112110347424784
   Tl   C
     2    12
Direct
  0.0000000000000000   0.0000000000000000   0.0000000000000000
```

```
0.5000000000000000  0.5000000000000000  0.5000000000000000
0.2500000000000000  0.0000000000000000  0.5000000000000000
0.7500000000000000  0.0000000000000000  0.5000000000000000
0.5000000000000000  0.2500000000000000  0.0000000000000000
0.5000000000000000  0.7500000000000000  0.0000000000000000
0.0000000000000000  0.5000000000000000  0.2500000000000000
0.0000000000000000  0.5000000000000000  0.7500000000000000
0.0000000000000000  0.2500000000000000  0.5000000000000000
0.0000000000000000  0.7500000000000000  0.5000000000000000
0.2500000000000000  0.5000000000000000  0.0000000000000000
0.7500000000000000  0.5000000000000000  0.0000000000000000
0.5000000000000000  0.0000000000000000  0.7500000000000000
0.5000000000000000  0.0000000000000000  0.2500000000000000
```

14. PO POSCAR files of $\boldsymbol{Fm\overline{3}m}$ - MC$_{10}$ with the highest T_c

NaC$_{10}$ at 80 GPa

```
1.000000
   0.00000000000000    3.17820000650000    3.17820000650000
   3.17820000650000    0.00000000000000    3.17820000650000
   3.17820000650000    3.17820000650000    0.00000000000000
Na C
1 10
Direct(11) [A10B1]
   0.00000000000000    0.00000000000000    0.00000000000000  Na
   0.75000000000000    0.75000000000000    0.75000000000000  C
   0.25000000000000    0.25000000000000    0.25000000000000  C
   0.61906999300000    0.61906999300000    0.14279002100000  C
   0.38093000700000    0.38093000700000    0.85720997900000  C
   0.14279002100000    0.61906999300000    0.61906999300000  C
   0.85720997900000    0.38093000700000    0.38093000700000  C
   0.61906999300000    0.61906999300000    0.61906999300000  C
   0.38093000700000    0.38093000700000    0.38093000700000  C
   0.61906999300000    0.14279002100000    0.61906999300000  C
   0.38093000700000    0.85720997900000    0.38093000700000  C
```

KC$_{10}$ at 80 GPa

```
1.000000
   0.00000000000000    3.20355010035000    3.20355010035000
   3.20355010035000    0.00000000000000    3.20355010035000
   3.20355010035000    3.20355010035000    0.00000000000000
K C
1 10
Direct(11)
   0.00000000000000    0.00000000000000    0.00000000000000  K
   0.25000000000000    0.25000000000000    0.25000000000000  C
   0.75000000000000    0.75000000000000    0.75000000000000  C
   0.38107997200000    0.85676008400000    0.38107997200000  C
   0.61892002800000    0.14323991600000    0.61892002800000  C
   0.38107997200000    0.38107997200000    0.38107997200000  C
   0.61892002800000    0.61892002800000    0.61892002800000  C
   0.85676008400000    0.38107997200000    0.38107997200000  C
   0.14323991600000    0.61892002800000    0.61892002800000  C
   0.38107997200000    0.38107997200000    0.85676008400000  C
   0.61892002800000    0.61892002800000    0.14323991600000  C   7
```

RbC$_{10}$ at 30 GPa

```
1.000000
   0.00000000000000    3.34086835414155    3.34086835414155
   3.34086835414155    0.00000000000000    3.34086835414155
   3.34086835414155    3.34086835414155    0.00000000000000
Rb C
```

1 10
Direct(11)

0.50000000000000	0.50000000000000	0.50000000000000	Rb
0.11811172600407	0.64566489598777	0.11811172600407	C
0.88188831099592	0.35433514101222	0.88188831099592	C
0.11811172600407	0.11811172600407	0.11811172600407	C
0.88188831099592	0.88188831099592	0.88188831099592	C
0.64566489598777	0.11811172600407	0.11811172600407	C
0.35433514101222	0.88188831099592	0.88188831099593	C
0.11811172600407	0.11811172600407	0.64566489598777	C
0.88188831099592	0.88188831099592	0.35433514101222	C
0.75000000000000	0.74999995000000	0.75000000000000	C
0.24999997500000	0.25000002500000	0.24999997500000	C

CsC_{10} at 10 GPa

1.000000

0.00000000000000	3.40064017700814	3.40064017700814
3.40064017700814	0.00000000000000	3.40064017700814
3.40064017700814	3.40064017700814	0.00000000000000

Cs C
1 10
Direct(11)

0.50000000000000	0.50000000000000	0.50000000000000	Cs
0.88246446203289	0.88246438803287	0.88246438803287	C
0.11753564896713	0.11753557496712	0.11753557496712	C
0.35260683590138	0.88246438803287	0.88246438803287	C
0.64739327509864	0.11753557496712	0.11753557496712	C
0.88246446203289	0.88246438803287	0.35260676190137	C
0.11753564896713	0.11753557496712	0.64739320109863	C
0.88246446203289	0.35260676190137	0.88246438803287	C
0.11753564896713	0.64739320109863	0.11753557496712	C
0.24999995000000	0.25000000000000	0.25000000000000	C
0.74999997500000	0.75000002500000	0.75000002500000	C

CaC_{10} at 120 GPa

1.000000

0.00000000000000	3.13377316221739	3.13377316221739
3.13377316221739	0.00000000000000	3.13377316221739
3.13377316221739	3.13377316221739	0.00000000000000

Ca C
1 10
Direct(11)

0.50000000000000	0.50000000000000	0.50000000000000	Ca
0.11969972510810	0.64090089867570	0.11969972510810	C
0.88030031189190	0.35909913832430	0.88030031189190	C
0.11969972510810	0.11969972510810	0.11969972510810	C
0.88030031189190	0.88030031189190	0.88030031189190	C
0.64090089867570	0.11969972510810	0.11969972510810	C
0.35909913832430	0.88030031189190	0.88030031189190	C
0.11969972510810	0.11969972510810	0.64090089867570	C
0.88030031189190	0.88030031189190	0.35909913832430	C
0.75000000000000	0.74999995000000	0.75000000000000	C
0.24999997500000	0.25000002500000	0.24999997500000	C

SrC_{10} at 140 GPa

1.000000

0.00000000000000	3.11326519704991	3.11326519704991
3.11326519704991	0.00000000000000	3.11326519704991
3.11326519704991	3.11326519704991	0.00000000000000

Sr C
1 10
Direct(11)

0.50000000000000	0.50000000000000	0.50000000000000	Sr
0.88033715497216	0.35898846108354	0.88033715497215	C
0.11966280802785	0.64101150191646	0.11966280802785	C
0.88033715497216	0.88033715497215	0.88033715497215	C
0.11966280802785	0.11966280802785	0.11966280802785	C
0.88033715497216	0.88033715497215	0.35898846108354	C

```
    0.11966280802785    0.11966280802785    0.64101150191646   C
    0.35898846108354    0.88033715497215    0.88033715497215   C
    0.64101150191646    0.11966280802785    0.11966280802785   C
    0.25000000000000    0.25000005000000    0.25000000000000   C
    0.75000002500000    0.74999997500000    0.75000002500000   C
```

BaC_{10} at 150 GPa

```
1.000000
    0.00000000000000    3.14656959556068    3.14656959556068
    3.14656959556068    0.00000000000000    3.14656959556068
    3.14656959556068    3.14656959556068    0.00000000000000
Ba C
1 10
Direct(11)
    0.50000000000000    0.50000000000000    0.50000000000000   Ba
    0.11932856912664    0.11932864312664    0.64201414462008   C
    0.88067131987336    0.88067139387336    0.35798589237992   C
    0.64201407062008    0.11932864312664    0.11932864312664   C
    0.35798581837992    0.88067139387336    0.88067139387336   C
    0.11932856912664    0.64201414462008    0.11932864312664   C
    0.88067131987336    0.35798589237992    0.88067139387336   C
    0.11932856912664    0.11932864312664    0.11932864312664   C
    0.88067131987336    0.88067139387336    0.88067139387336   C
    0.75000005000000    0.75000000000000    0.74999995000000   C
    0.25000002500000    0.24999997500000    0.25000002500000   C
```

ScC_{10} at 0 GPa

```
1.000000
    0.00000000000000    3.33533467072833    3.33533467072833
    3.33533467072833    0.00000000000000    3.33533467072833
    3.33533467072833    3.33533467072833    0.00000000000000
Sc C
1 10
Direct(11)
    0.50000000000000    0.50000000000000    0.50000000000000   Sc
    0.11896806683587    0.64309587349240    0.11896806683587   C
    0.88103197016413    0.35690416350760    0.88103197016413   C
    0.11896806683587    0.11896806683587    0.11896806683587   C
    0.88103197016413    0.88103197016413    0.88103197016413   C
    0.64309587349240    0.11896806683587    0.11896806683587   C
    0.35690416350760    0.88103197016413    0.88103197016413   C
    0.11896806683587    0.11896806683587    0.64309587349240   C
    0.88103197016413    0.88103197016413    0.35690416350760   C
    0.75000000000000    0.74999995000000    0.75000000000000   C
    0.24999997500000    0.25000002500000    0.24999997500000   C
```

AlC_{10} at 80 GPa

```
1.000000
    0.00000000000000    3.19245004655000    3.19245004655000
    3.19245004655000    0.00000000000000    3.19245004655000
    3.19245004655000    3.19245004655000    0.00000000000000
Al C
1 10
Direct(11)
    0.00000000000000    0.00000000000000    0.00000000000000   Al
    0.25000000000000    0.25000000000000    0.25000000000000   C
    0.75000000000000    0.75000000000000    0.75000000000000   C
    0.38111001300000    0.38111001300000    0.85666996100000   C
    0.61888998700000    0.61888998700000    0.14333003900000   C
    0.85666996100000    0.38111001300000    0.38111001300000   C
    0.14333003900000    0.61888998700000    0.61888998700000   C
    0.38111001300000    0.38111001300000    0.38111001300000   C
    0.61888998700000    0.61888998700000    0.61888998700000   C
    0.38111001300000    0.85666996100000    0.38111001300000   C
    0.61888998700000    0.14333003900000    0.61888998700000   C
```

GaC_{10} at 80 GPa

```
1.000000
```

```
    0.00000000000000    3.19234991075000    3.19234991075000
    3.19234991075000    0.00000000000000    3.19234991075000
    3.19234991075000    3.19234991075000    0.00000000000000
Ga C
1 10
Direct(11)
    0.00000000000000    0.00000000000000    0.00000000000000   Ga
    0.75000000000000    0.75000000000000    0.75000000000000   C
    0.25000000000000    0.25000000000000    0.25000000000000   C
    0.61879998400000    0.61879998400000    0.14360004800000   C
    0.38120001600000    0.38120001600000    0.85639995200000   C
    0.14360004800000    0.61879998400000    0.61879998400000   C
    0.85639995200000    0.38120001600000    0.38120001600000   C
    0.61879998400000    0.61879998400000    0.61879998400000   C
    0.38120001600000    0.38120001600000    0.38120001600000   C
    0.61879998400000    0.14360004800000    0.61879998400000   C
    0.38120001600000    0.85639995200000    0.38120001600000   C
```

LaC_{10} at 180 GPa

```
1.000000
    0.00000000000000    3.09192437471908    3.09192437471908
    3.09192437471908    0.00000000000000    3.09192437471908
    3.09192437471908    3.09192437471908    0.00000000000000
La C
1 10
Direct(11)
    0.50000000000000    0.50000000000000    0.50000000000000   La
    0.88000499047423    0.88000491647423    0.88000491647423   C
    0.11999512052577    0.11999504652577    0.11999504652577   C
    0.35998525057730    0.88000491647423    0.88000491647423   C
    0.64001486042270    0.11999504652577    0.11999504652577   C
    0.88000499047423    0.88000491647423    0.35998517657730   C
    0.11999512052577    0.11999504652577    0.64001478642270   C
    0.88000499047423    0.35998517657730    0.88000491647423   C
    0.11999512052577    0.64001478642270    0.11999504652577   C
    0.24999995000000    0.25000000000000    0.25000000000000   C
    0.74999997500000    0.75000002500000    0.75000002500000   C
```
